# Nesting box imager: Contact-free, real-time measurement of activity, surface body temperature, and respiratory rate applied to hibernating mouse models

**DOI:** 10.1371/journal.pbio.3000406

**Published:** 2019-07-24

**Authors:** Nathaniel E. Kallmyer, Han Jong Shin, Ethan A. Brem, William J. Israelsen, Nigel F. Reuel

**Affiliations:** 1 Iowa State University, Ames, Iowa, United States of America; 2 University of Texas Southwestern Medical Center, Dallas, Texas, United States of America; University of Sussex, UNITED KINGDOM

## Abstract

Noncontact methods to measure animal activity and physiology are necessary to monitor undisturbed states such as hibernation. Although some noncontact measurement systems are commercially available, they are often incompatible with realistic habitats, which feature freely moving animals in small, cluttered environments. A growing market of single-board computers, microcontrollers, and inexpensive sensors has made it possible to assemble bespoke integrated sensor systems at significantly lower price points. Herein, we describe a custom-built nesting box imager (NBI) that uses a single-board computer (Raspberry Pi) with a passive infrared (IR) motion sensor, silicon charge-coupled device (CCD), and IR camera CCD to monitor the activity, surface body temperature, and respiratory rate of the meadow jumping mouse during hibernation cycles. The data are logged up to 12 samples per minute and postprocessed using custom Matlab scripts. The entire unit can be built at a price point below US$400, which will be drastically reduced as IR (thermal) arrays are integrated into more consumer electronics and become less expensive.

## Abstract 2

By coupling single-board computers with increasingly available, inexpensive sensors and cameras, it is possible to construct custom, low-cost animal habitats that monitor undisturbed activity and physiological parameters with real-time resolution.

## Introduction

Many biological phenomena of interest occur in closed systems that are inconvenient or impossible to measure with traditional wired sensors (e.g., thermocouples, pH probes, pressure sensors). One such environment is the enclosed nest site of a free-moving animal, whether in a laboratory setting or in the field. There is significant biologic interest in monitoring the activity and physiological indicators—such as body temperature and respiration rate—of undisturbed animals with temporal resolution [1], but noncontact options for such measurements during sleep/wake cycles or hibernation have been limited. Historically, the body temperature of small hibernators was measured using thermocouples inserted orally or rectally [2,3], and respiratory rate was determined via timed visual counting of breaths [4]. More recently, surgically implantable or attachable telemeters or loggers have come into common use for measuring conditions such as core body temperature and heart rate in captive and free-ranging animals [5–8].

Current methods for monitoring animal activity, respiration, and temperature generally employ commercially available systems designed for rodents in biomedical laboratory settings (summarized in S1 Table). Animal activity–monitoring options typically include infrared (IR) beam-breaking sensors, running wheels, video-based motion tracking, and/or force plate actometers [9–12]. These monitoring systems usually require an uncluttered cage or direct interaction of the animal with the running wheel or force plate and are not amenable to monitoring in enclosed or remote environments. Respiration of small, unrestrained animals is most often measured using a whole-body plethysmograph [13], in which the animal is placed in a dedicated instrument chamber that is not easily integrated into a nest environment. Recent video-based methods for monitoring respiration have required immobilization of the subject [14,15]. Surface body temperature can be determined in a noncontact fashion by thermal imaging or video systems, which require an unobstructed view of the subject [16]. Adoption of such tools has been limited by their size and the proprietary nature of closed systems, which limits convenient data logging or synchronization to other system sensors; moreover, the complete system (sensors, controller, and software) drives up the cost, typically to a quoted purchase in the US$5,000–US$50,000 range. Outside of a laboratory setting, recording of animals in nests or burrows requires direct visual observation by the researcher or the use of visual or IR cameras recording images or videos [17].

Recent advances in single-board computers (e.g., Raspberry Pi), microcontrollers (e.g., Arduino), and inexpensive, high-quality cameras from cell-phone manufacturers have made it possible to create high-precision, custom imagers at a low price point, such as a €100 fluorescent microscope or the US$1 foldscope (US$40 at retail) [18,19]. Some of these low-cost components, such as IR camera modules, have been used to build animal imagers, such as bird box cameras and wildlife imagers. Moreover, there is a greater movement to design “frugal science” instruments to democratize the process of scientific inquiry to all and make everyone a “citizen scientist” [20].

Herein, we demonstrate a new “frugal science” device that, for the first time, integrates three IR camera/detectors and open-source algorithms to provide a nesting box imager (NBI) that monitors animal activity, respiration, and temperature without making contact or disturbing the small animal. At the time of writing, a single unit costs US$400 to build with off-the-shelf parts and would dramatically drop in price at scale. The IR sensors include (1) a passive IR (PIR) motion detector to track levels of activity, (2) a near-IR camera to image the animal and track respiration rate, and 3) a thermal IR camera to track the animal surface temperature. Herein, we detail the design of the open-source NBI and show its utility with a hibernating rodent model, the meadow jumping mouse (*Zapus hudsonius*). The NBI design presented here overcomes some limitations of existing animal monitoring techniques—namely, improved time and spatial resolution of measurement in the nest. This instrument allows for customized, inexpensive, contact-free measurement of animals in a small environment, with temporal resolution of up to 12 samples per minute over an 8-hour time frame (using a 32 GB flash drive); this recording duration can be dramatically increased by reducing the resolution of the video used to calculate the respiration rate or by collecting this respiration data at less-frequent intervals.

## Results

### Nesting box design for small rodents

**Fig 1:**
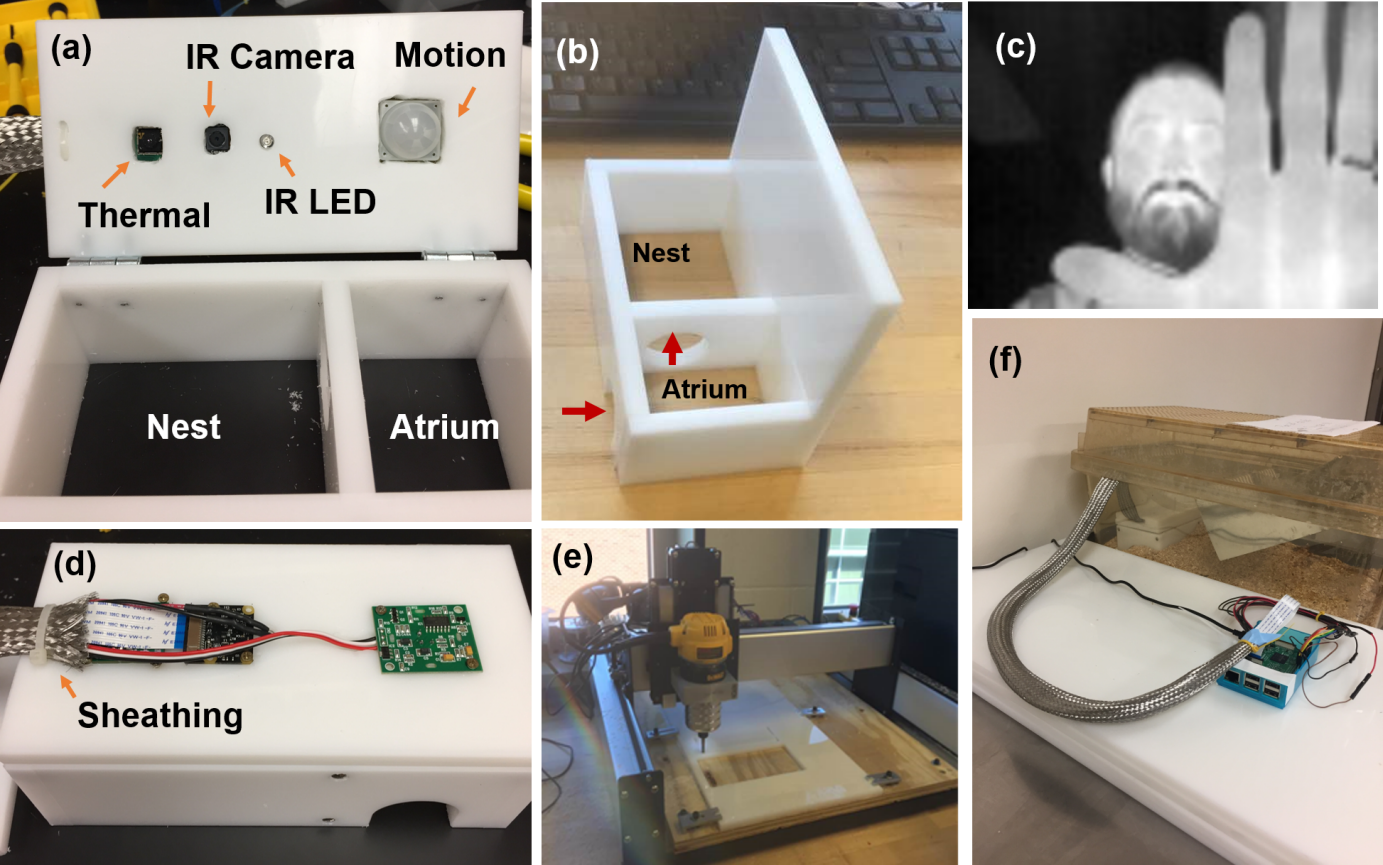
NBI design. (a) NBI includes an entrance atrium monitored by a motion sensor and a nest compartment monitored by a thermal and IR camera (illuminated by an IR LED). (b) Path of mouse to the nest compartment. (c) Representative image captured from thermal camera showing low, 80-×-60-pixel resolution. (d) Sheathing to protect wires from mice. (e) Mill used to cut plastic frame. (f) NBI placed in cage showing single-board computer (Raspberry Pi) to execute imaging programs and record data (note this current NBI system is powered by a wall plug). IR, infrared; LED, light-emitting diode; NBI, nesting box imager.

The nesting box was designed after two-chambered nest boxes used for the husbandry of lesser Egyptian jerboas, a much larger but related rodent species [21]. The current boxes were designed to fit inside the cage size used for laboratory housing of meadow jumping mice, with the footprints of the outer atrium and nesting room at 9 × 7 cm and 9 × 12 cm, respectively (Fig 1; design in S1 and S2 Figs). We observed that this design was very effective with this species in that all mice studied chose to sleep inside the dark, back room of the box (nest chamber). The atrium was built with circular entrances to the exterior and nesting room (both 3.2 cm diameter). With the addition of a motion detector, the atrium functioned as a controlled environment to track the periods of high activity of the mouse. An 80-×-60-pixel long-wave IR imager (FLIR Lepton), a 1920-×-1080-pixel, 30-frames-per-second (fps) near-IR camera (Raspberry Pi NoIR camera), and an 890-nm IR illumination source (light-emitting diode [LED] SparkFun) were positioned above the nest chamber. Illumination from the IR LED is outside the visible range of mammals, including rodents [22]. The combination of the IR camera and 890-nm IR illumination thus allowed image capture, regardless of outside illumination, in a manner that did not disturb the subject. The bottom of the nesting box was left open to allow for positioning on top of the natural bedding present in the mouse cage.

The box lid was hinged to allow for ready access for cleaning. Because of the propensity of rodents to gnaw on objects in their environment, a stainless-steel sheathing (Techflex SSL0.75SV) was installed (Fig 1D) in addition to an enclosed cover (shown complete in Fig 1F) to prevent any access to wires or electronic components. The nest box was constructed of 1-cm-thick high-density polyethylene milled using a desktop computer numerical control (CNC) mill (Shapeoko 3—see Materials and methods below). The full parts list is provided in S2 Table, and a hookup guide is provided in S3 Table and S4 Fig.

### Motion measurements for animal activity

**Fig 2:**
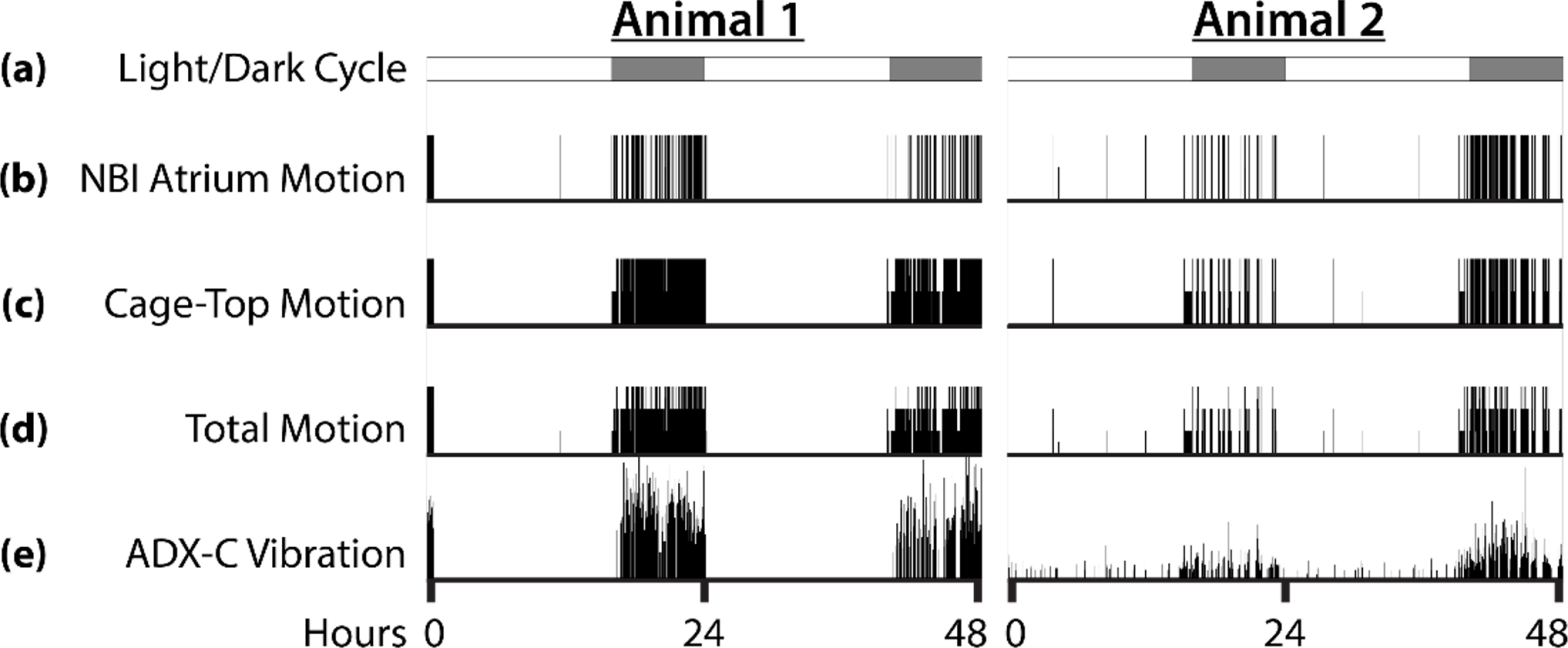
Actograms showing activity from two separate animals over periods of 48 hours. The x-axis represents time, and motion is reported in arbitrary units on the y-axis. (a) Schematic showing light/dark cycle. White bars indicate light, and gray bars indicate dark. The animals were held under “summer” conditions of 16 hours light and 8 hours dark. (b) Activity data recorded from NBI atrium PIR motion sensor. (c) Activity data recorded from two cage-top PIR motion sensors. Output from each sensor was weighted equally and summed. (d) Activity data combined from NBI motion sensor and two cage-top motion sensors. Activity from each of the three PIR sensors was weighted equally and summed. (e) Reference activity recorded from a Sable Systems International ADX-C pad placed under the cage. This method of activity recording detects all vibration in the cage caused by animal movement. The data underlying this figure can be found in S1 Data. NBI, nesting box imager; PIR, passive infrared.

The atrium is monitored by a PIR motion sensor, which detects changes in IR light emitted by a moving mouse. This sensor runs on an independent script (S1 Code) that records the time points of motion events. In this manner, the activity and exit frequency of the mouse can be quantified. To determine the utility of the NBI motion sensor, we compared activity recordings from the NBI to a commercial activity-monitoring system. Simultaneous recordings were obtained from the NBI motion sensor, an under-cage activity-monitoring pad that detects vibrations caused by mouse motion (model number ADX-C, Sable Systems International), and from two PIR sensors mounted inside the cage lid. The ADX-C recordings provide a measure of total animal activity anywhere in the cage, the cage-top PIR sensors capture motion outside of the nest box, and the NBI sensor captures motion inside the nest box atrium. Representative actograms of 48-hour recordings from two mice are shown in Fig 2. Meadow jumping mice are crepuscular/nocturnal, and the activity data show little to no activity outside the nest box during the light cycle. In some cases, the animal appears to have triggered the NBI motion sensor in the atrium but not left the nest box during the day. The actograms plotted from ADX-C vibration data display qualitative agreement with total PIR activity (NBI + cage-top PIR sensors) and with the NBI and cage-top activity data plotted alone. To provide a quantitative comparison of the recording methods, 24-hour periods of activity were integrated over time (S1 Text and S3 Fig) and compared using Pearson’s correlation coefficient (*n* = 9, 3 periods from 3 animals). The NBI motion data, cage-top motion data, and total PIR motion data are highly correlated with the ADX-C vibration data (S4 and S5 Tables; mean correlations: r = 0.979, r = 0.978, r = 0.983, respectively), suggesting that NBI activity recordings alone could serve as a proxy measure of overall daily activity in this species.

### Surface body temperature measurements

**Fig 3:**
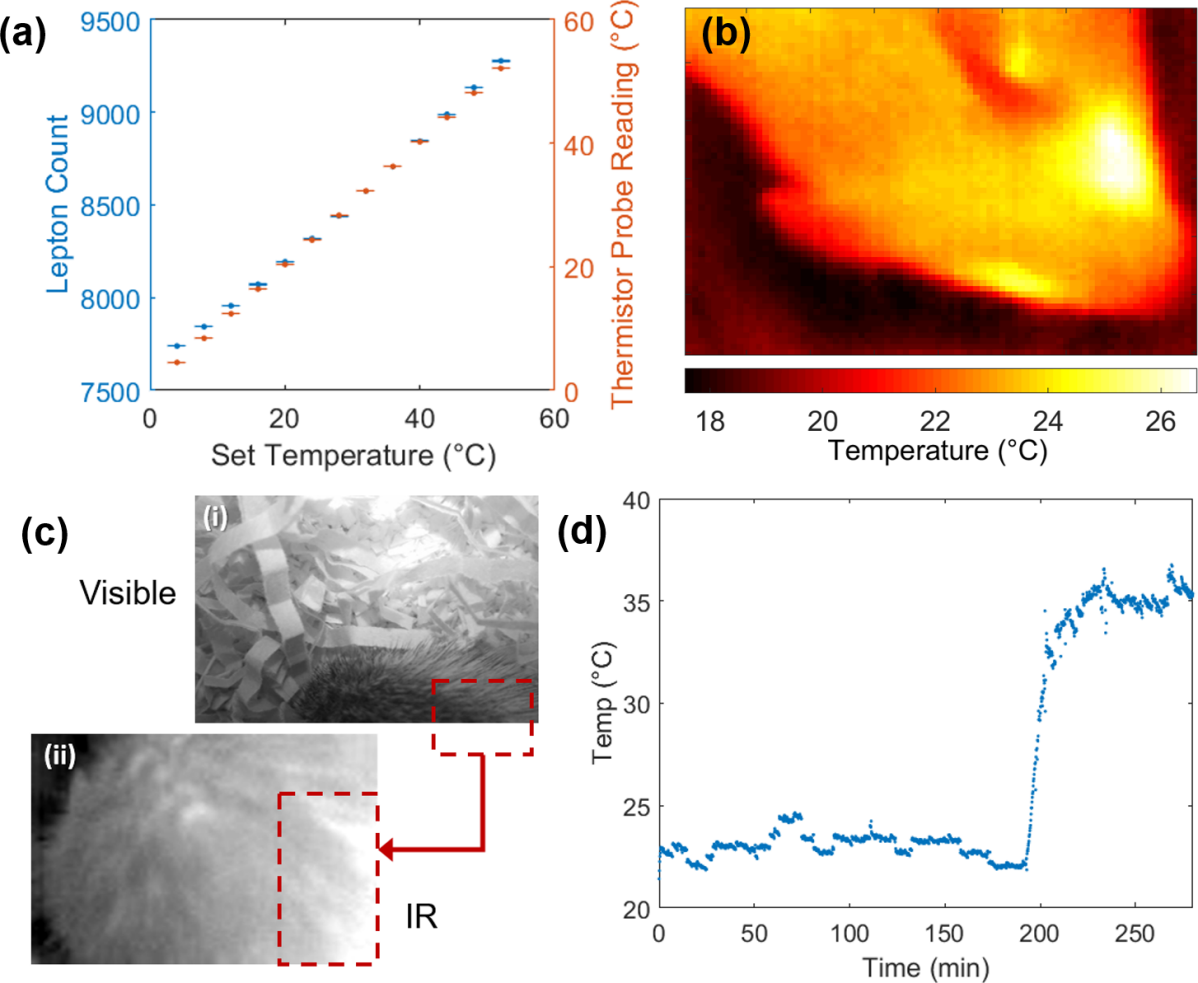
Noncontact thermal imaging of mouse models in NBI. (a) Calibration curve of IR camera (Lepton units) to temperatures observed on thermal cycler and comparison to temperatures measured by thermistor probe (*n* = 6 measurements for each condition, one standard deviation shown as error bars). (b) Heat map of mouse head using calibration curve found in (a). (c) Representative images from NBI of a nested mouse—visible image of mouse back (i) and corresponding thermal, IR image of mouse (ii); red box shows region of analysis with 10% top pixels. (d) Surface body temperature of a meadow jumping mouse that had entered torpor and cooled to room temperature (approximately 22°C). Spontaneous arousal from torpor is detected as a rapid increase in surface body temperature at around 200 seconds. The data underlying this figure can be found in S2 Data. IR, infrared; NBI, nesting box imager.

Surface body temperature correlates to metabolic state and was an additional desired measure for this hibernating mouse model. A small, inexpensive thermal IR camera (FLIR Lepton) was positioned above the nesting chamber, and the thermal signature of the mouse was tracked to determine temperature over time. These camera units were calibrated to surface temperatures of a thermal cycler block set at different temperature points (Materials and methods) and were validated using a commercial thermistor probe (Fig 3A). For further validation, we used the thermistor probe on proxy models (human hand) and five mice at different temperatures and compared readings from the IR camera (using the linear transfer function found in Fig 3A) to the temperature measured by the thermistor (Table 1 and S2 Data). The average deviation between the two methods was found to be 0.74°C. Spatial temperature maps of the mouse can be generated (Fig 3B); however, in the NBI, the mouse can shift position and partially cover itself with bedding. To overcome these movements and account for large portions of the images containing bedding and other background pixels, only the brightest 10% of the 4,800 pixels were averaged to calculate the camera measurement of mouse surface temperature (“Lepton units”) (Fig 3C). In this manner, the dynamic changes in animal temperatures can be monitored without disturbing the animal, such as the temperature change of a mouse coming out of torpor (Fig 3D).

**Table 1:** Validation of temperature measurements. Average of *n* = 5 images for each condition (all in °C).

Temperature	Hand warm	Hand cold	Mouse 1	Mouse 2	Mouse 3	Mouse 4	Mouse 5	Avg
TC	33.88	25.25	34.60	33.64	33.74	33.38	34.97	
tm	34.04	25.67	33.96	33.37	34.09	32.81	36.23	
Δ |(TC − tm)|	0.45	1.63	0.64	0.28	0.35	0.58	1.26	0.74
St. dev (*n* = 5) TC	0.03	0.11	0.42	0.28	0.30	0.88	0.50	0.36

Abbreviations: Avg, average; St. dev, standard deviation; TC, thermal camera; tm, thermistor

### Respiratory rate measurements

**Fig 4:**
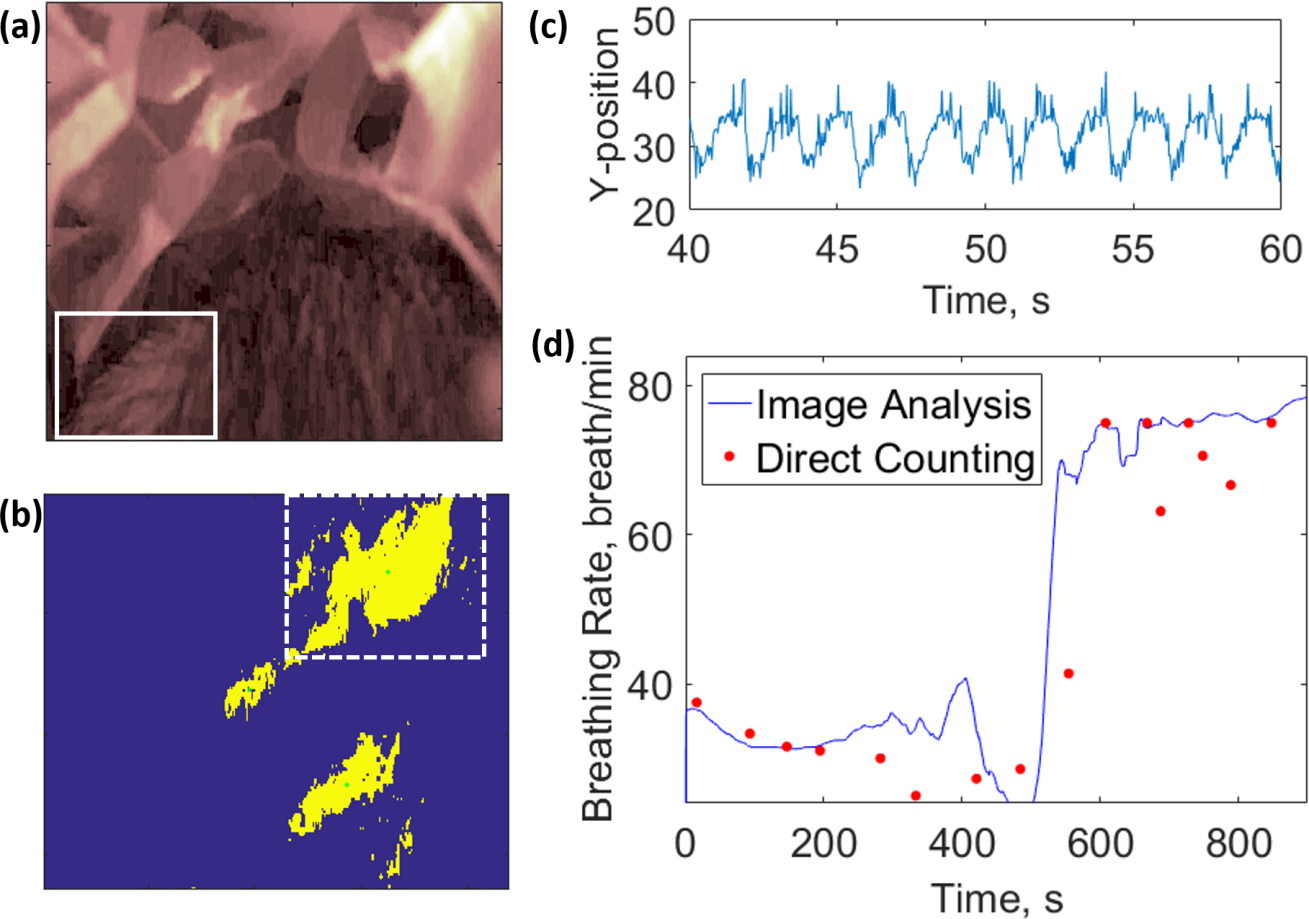
Respiratory monitoring of a single mouse exiting torpor. (a) Visible spectrum image of mouse constructed with green pixel data. Solid white box indicates manually selected region of interest. (b) Binary-converted (1-bit) image of region of interest (solid white box from [a]). Green pixels indicate followable features recognizable to the Matlab centroid function. Dashed white box encloses shape followed for breathing analysis. (c) Y-pixel position of object of interest with respect to time. (d) Mouse breathing frequency with respect to time. Solid blue line indicates frequency calculated based on a Fourier transform of (c). Red dots indicate breathing frequencies measured by direct video observation and breath counting. The data underlying this figure can be found in S3 Data.

Mouse breathing frequency was extracted from video data collected via the near-IR camera (Raspberry Pi NoIR, static images or high-definition [HD] video) (Fig 4A). An algorithm (Materials and methods) was designed to track X- and Y-motion of regions of interest (Fig 4B) to map the periodic motion associated with breathing (Fic 4C). With a moving time window, a Fourier transform was used to convert the time domain data to a frequency domain. The respiratory rate was found as the most prominent peak of this frequency data. The video analysis algorithm was first validated by capturing simulated respiratory frequencies using an elastic proxy mouse and was found to be in agreement in the 30 to 240-breaths-per-minute range (summarized in S3 Text and shown in S5 and S6 Figs). This algorithm was then applied to footage of real mice captured from the NBI. One interesting example is that of a mouse exiting torpor, in which we observed a 2-fold increase in breathing frequency within 10 minutes, which was also validated using the established method of visual inspection of the footage (Fig 4D).

## Discussion

The desired features of the NBI—namely, contact-free measurement of activity, temperature, and respiration of small animal models in an inexpensive, small form factor for use in the vivarium—were obtained. We found that the meadow jumping mice preferred to sleep in the nest chamber and that the data streams could be conveniently recorded on a flash drive and transferred to a computer for postprocessing after completion of the experiment. Uncompressed video data, produced at approximately 4 GB per hour, limit recordings to a maximum of 8 hours with a standard, 32 GB USB drive. In the current setup, it is powered by a wall plug, so there is no limitation on battery life. A few design elements were included to ensure the robustness of the box against excessive mouse mastication. No electronic components were accessible to the animals, and we never observed any attempts to gnaw on the stainless-steel sheathing enclosing the wires. The top cover for the electronics successfully preserved the equipment and prevented any contact when the animal was active on top of the nest box.

For activity monitoring, we found the PIR motion sensor in the NBI atrium provided data that correlated with daily animal activity data from a commercial activity-monitoring system. Deployment of the atrium PIR sensor provides an example of how a sensor in an enclosed space can provide a direct measure of a specific activity—in this case, nest box entry/exit and time spent in the atrium. Whereas the reference data from the cage-top motion sensors were recorded using commercial equipment, the modularity of the Python scripts and available Raspberry Pi general-purpose input/output (GPIO) pins allow for addition of additional PIR sensors to the NBI. Such sensors could be used as cage-top sensors or to meet other, specific needs of the research.

Both the IR and thermal cameras were found to have limitations when obstructed by the nest bedding. Occasionally, the nesting behavior of the mouse covers the camera with nest material; however, by simply lifting the lid and moving it back in place, data can continue to be collected. This could be improved in subsequent designs by selecting less voluminous nesting material and would likely not be a problem when recording species, such as laboratory mice, that build less elaborate nests. We also found that in cold chamber conditions, condensation could build up on the thermal camera lens if care was not taken to remove humid air from the environment. Although some data from thermal cameras affected by condensation still appeared to be valid, we found that, eventually, the camera performance would suffer and require replacement. The temperatures of mice in torpor and active state (23 and 35°C, Fig 3D) also correlate well with data obtained using handheld IR thermometers (S6 Fig), which require disturbing the nest box to make measurements. The IR camera approach also has the added benefit of averaging the temperatures over a larger area versus the point measurements of thermistors, which can vary based on where the probe is positioned for measurement.

Respiratory motion was determined by tracking the position of a visible spot on the fur of the meadow jumping mouse (Fig 4A). This method of motion tracking was found to reliably report movement frequencies between 0.5 and 4 Hz, or 30–240 breaths per minute. At lower frequencies, this algorithm exhibited a positive bias, which could be reduced by widening the windows of time used in Fourier transform analysis (S7 Fig). Although the need for a trackable feature would seemingly limit the ability to monitor different mice, consistent, higher-contrast spots can be artificially created through use of a dye or indelible ink. Reliable tracking requires selection of a region of interest sufficiently large to capture the entire range of motion of a selected feature, a range which may be determined by watching the first 30 seconds of video footage. Although the size of this region depends on the camera angle and distance from the mouse, the user may more intuitively select region bounds by clicking on a reproduced image (via Matlab “ginput” function, S2 Code). A limitation of this algorithm is its susceptibility to additional recognizable features within the selected region of interest that confound image recognition. This interference can be mitigated by using stricter pixel recognition criteria to retain fewer followable shapes. The effects of extraneous features may also be reduced by limiting the region of interest to the range of motion for a single feature, minimizing the chance of additional features appearing. For situations in which movement amplitude is too large to use a small region of interest, a median filter may be applied to frequency data to remove artifact spikes or drops associated with tracking of extraneous features. Although there exist many methods to reduce artifacts, measurements are still limited to periods of relatively stationary behavior during which movement is limited to slight repositioning. Another limitation to this algorithm is that, with a standard computer (<24 GB RAM), the computation time is approximately as long as the analyzed video. This time may be reduced substantially by using lower-resolution video files, which in turn would also allow for longer recording times.

## Next steps

The meadow jumping mouse was a good introductory species to test the NBI, as it required a small, tight design. For larger animal model species, it would be straightforward to expand the design or include multiple cameras to provide additional angles of observation. For field applications, the relatively low power consumption of the NBI would allow long battery-powered runtimes of a weather-proofed version of the design. A quick analysis of part costs reveals that two-thirds of the unit cost is the thermal camera. As these small thermal camera modules are adapted into consumer electronics (as HD cameras were in cell phones), we expect a large price decrease in this more expensive sensor. However, at the current price point of US$400 per NBI without bulk-part savings, this still can be a useful tool in many applications ranging from large-scale animal monitoring in research vivariums to “citizen science” applications at home and primary schools.

## Materials and methods

### Construction of nest box frame

Components of the nest box frame were prepared by cutting a 9.5-mm (3/8 inch) sheet of white high-density polyethylene with a 3D carbide cutter (ShapeOko 3) into five separate elements. These elements were attached by epoxy glue to form a roofed, floorless, two-room structure (detailed plans in S2 Fig). A circular aperture was cut into the central dividing wall to allow transport between rooms, and an arched gap was cut into the outer wall to allow entry and exit. Four holes were cut into the roof to accommodate electrical components including an IR LED, a thermal camera, a silicon detector camera with no IR filter, and a motion sensor. These electrical components were fastened with adhesive and/or screws.

### Sensor wiring

Sensing and working components of the NBI were controlled by a single-board computer (Raspberry Pi version 3). The thermal camera (FLIR Lepton) was powered with 5 V from the Pi and communicated with the Pi by GPIO pins. A visible spectrum camera enhanced to near-IR light (Pi NoIR version 2) was connected to the Pi by ribbon cable. The mouse was illuminated by an 890-nm IR LED, which was both powered and triggered by a 3-V GPIO pin connected to a 220-Ω resistor, which acted as a voltage divider. A red and green LED were similarly connected to indicate the on/off and data collection state of the vivarium. A PIR motion sensor was powered with 3.3 V from the Pi and also connected to GPIO pins. A shutoff button was also connected to GPIO pins to save video data prior to shut down. A hookup guide is available in S4 Fig and S3 Table.

### Control software

The NBI sensors were controlled by Python scripts (S1 Code). The thermal camera was set to collect an image with 1-second intervals and save each image to a separate text file. The near-IR camera was set to continuously record video data and save this data to an h264 video file after input from the shutdown button. The motion sensor was set to operate continuously and record a time stamp to a text file following an observed motion event. Execution of these sensing functions was automated by a launch script. Libraries and updates installed are listed in S6 Table.

### Animal observations

This study was conducted according to the recommendations in the Guide for the Care and Use of Laboratory Animals of the National Institutes of Health. Animal work described in this manuscript has been approved and conducted under the oversight of the University of Texas Southwestern Institutional Animal Care and Use Committee (protocol #2015–101240) and the Massachusetts Institute of Technology Committee on Animal Care (protocol #0514-048-17). Individual meadow jumping mice were housed singly in polycarbonate cages (46 cm × 24 cm × 15 cm). Each cage was provided with corncob bedding and nesting material (Enviro-dri paper strips), and standard enrichment included a translucent polycarbonate igloo or noninstrumented nest box and a wood block or stick. To allow recording, the standard shelter was replaced with the NBI, and recordings were typically begun following one day of acclimation. Recordings were performed at room temperature (approximately 22°C).

### Data analysis

Data are analyzed with custom Matlab scripts, available in S2 Code. The motion sensor data were recorded as a delimited text file (“motion.txt”), which begins with a header comment noting the experiment start time and then a single-column vector of all time points when the motion sensor is activated. The Matlab script “MouseMove.m” is used to parse this vector and convert the string entries to time values (seconds after experiment start). These can then be plotted, such as the activity diagrams in Fig 2.

The thermal IR data were recorded as delimited text files of the raw Lepton camera units (60 × 80 matrix) for each time step. These files were then analyzed by the “ThermoVec.m” function to determine the time point from the file name and to take the average pixel value from the top 10% of the pixels. The time points and average pixel values were then saved as a *.csv file for plotting by “plotTherm.m”. This script converts the average pixel value to an actual temperature using the calibration data (Fig 3A) fit with a linear model. The calibration data can be readily obtained for any new camera by first taking images of an object with set surface temperatures (e.g., hot plate, thermal mixer, PCR thermalcycler).

The IR camera data were processed with a custom Matlab script (“MouseVideoTest.m”) to extract the breathing rates over time. In brief, the green pixel data (highest contrast of the RGB camera data) were converted to a binary image (Fig 4B) based on user-selected pixel intensity threshold, and a shape recognition algorithm (centroid function Matlab) was used to track regions of interest on the mouse (Fig 4C). A Fourier transform was applied to a moving timeframe, and the respiratory rate was determined as the most prominent frequency peak in the transformed data. Detailed analysis instructions are provided in S3 Text.

## Ethics statement

This study was conducted according to the recommendations in the Guide for the Care and Use of Laboratory Animals of the National Institutes of Health. Animal work described in this manuscript has been approved and conducted under the oversight of the University of Texas Southwestern Institutional Animal Care and Use Committee (protocol #2015–101240) and the Massachusetts Institute of Technology Committee on Animal Care (protocol #0514-048-17).

## Supporting information

S1 Text Summary of mouse motion analysis used in comparison of nesting box imager PIR motion detector to a commercial system. PIR, passive infrared.
(PDF)

S2 Text Description of calibration tests for respiratory motion tracking using model system in orbital shaker. (PDF)

S3 Text Instructions for running video analysis to obtain respiratory rate measurements and minimizing noise in the algorithm’s output. (PDF)

S1 Fig Two-room nesting box imager without embedded sensors prepared from 3/8-inch HDPE and affixed with epoxy. HDPE, high-density polyethylene.
(PDF)

S2 Fig (Right) Dimensions of nesting box imager, cut out of 3/8-inch white HDPE panels. A = top plate; B = side plate (no hole); C = side plate, entrance hole; D = side walls (×2); and D = interior wall between atrium and nesting room. (Left) Cut out image from Shapeoko Carbide cutter, on 1/4-inch grid spacing. Additional cuts are made into part A to embed sensors. HDPE, high-density polyethylene.
(PDF)

S3 Fig Normalized plots of integrated motion data from the different motion sensing methods compared in this paper. Each panel depicts 24 hours of motion starting with onset of dark cycle (*n* = 9 days, 3 days each from three different animals). Individual lines represent activity as recorded via ADX-C Vibration, NBI Atrium Motion, Cage-top PIR Motion, and Total PIR Motion, which is the sum of the Cage-top PIR Motion and NBI Atrium Motion. Individual animals have different patterns of activity, and the PIR-based motion sensor data generally correspond well with the commercial ADX-C vibration sensing pad. The data underlying this figure can be found in S4 Data. NBI, nesting box imager; PIR, passive infrared.
(PDF)

S4 Fig Hookup of three diodes, motion sensor, and shutdown button to Raspberry Pi. LEDs were connected in series with 220-Ω resistors to limit current. LED, light-emitting diode.
(PDF)

S5 Fig Proxy “mouse” used for respiratory rate calibration and analysis. Dashed boxes enclose three different regions used in image analysis.
(PDF)

S6 Fig Respiratory rate data collected by analyzing mouse model using all X- and Y-motion data from each region (a) (*n* = 6) and using motion data after removing outliers (b) (*n* = 4). Shaded regions indicate a single standard deviation. The data underlying this figure can be found in S5 Data.
(PDF)

S7 Fig Temperatures of mice either awake or in torpor at room temperature using a handheld IR thermometer. Surface temperatures were determined by periodic measurements of a group of seven mice over 23 days using an IR thermometer. Ambient temperatures were determined as the temperature of the bedding at the same time that the mice were measured (not all paired). Each mouse and bedding was only measured once on the day that they were measured. Error bars indicate single standard deviation calculated for 15, 13, and seven measurements for ambient, torpid, and awake temperatures, respectively. The data underlying this figure can be found in S6 Data. IR, infrared.
(PDF)

S8 Fig Effect of timeframe size and filtering on frequency output accuracy and stability. Different plots indicate algorithm outputs with timeframes (size of time window used in Fourier transform) of 13 seconds (a), 26 seconds (b), 40 seconds (c), and 66 seconds (d). Solid black lines indicate algorithm output using only a rolling average smoothing filter (average over 800 timepoints, 26 seconds). Solid blue lines are smoothed with a median filter prior to rolling average smoothing. Red dots are measurements collected by directly observing mouse footage. The data underlying this figure can be found in S3 Data.
(PDF)

S9 Fig Manual selection of region for analysis for mouse video used in respiratory rate fidelity analysis. (Left) Upper-left bound of region C (shown in S5 Fig—“mouse” left ear), selected first. (Right) Lower-right bound of region C, selected second.
(PDF)

S1 Table Review of current methods of animal monitoring. (PDF)

S2 Table Parts list for nesting box imager. (PDF)

S3 Table Raspberry Pi GPIO pin hookup. The red highlighted boxes connect to the thermal camera. The orange highlighted boxes connect to the PIR motion sensor. The gray, light-red, and green highlighted boxes connect to the NIR, red, and green LEDs, respectively. The blue highlighted boxes connect to the safe shutoff button. GPIO, general-purpose input/output; LED, light-emitting diode; NIR, nesting box imager; PIR, passive infrared.
(PDF)

S4 Table Pearson correlation coefficients between a commercial motion sensor and PIR motion sensors secured in different positions. PIR, passive infrared.
(PDF)

S5 Table Summary of Pearson correlation coefficients between PIR sensors and commercial motion sensor. PIR, passive infrared.
(PDF)

S6 Table Libraries installed to Raspberry Pi following update to preinstalled NOOBS) operating system. NOOBS, New Out of the Box Software.
(PDF)

S1 Code Python scripts for thermal camera, motion sensor, button trigger, and motion data processing. (PDF)

S2 Code Matlab scripts used for analysis of motion data, thermal imaging data, and respiratory motion data. (PDF)

S3 Code Python script used to prepare data used to compare PIR motion sensors to commercial motion sensors. PIR, passive infrared.
(PDF)

S1 Data Raw data used for motion tracking of the two mice in Fig 2. (XLSX)

S2 Data Thermal calibration and mouse surface temperature data used for Fig 3 and Table 1. (XLSX)

S3 Data Raw feature position data, calculated respiratory rates, and directly observed respiratory rates used for Fig 4 and S3 Text and S8 Fig. (XLSX)

S4 Data Raw activity data used for activity study of three mice over 3 days in S1 Text and S3 Fig and S4 and S5 Tables. (XLSB)

S5 Data Respiratory rates from model mouse system collected by direct observation and video analysis used in calibration of respiratory rate measurement in S2 Text and S5 Fig. (XLSX)

S6 Data Handheld IR temperature data collected for S6 Fig. IR, infrared.
(XLSX)

## Abbreviations

CCD: charge-coupled device
CNC: computer numerical control
fps: frames per second
GPIO: general-purpose input/output
HD: high-definition
IR: infrared
LED: light-emitting diode
NBI: nesting box imager
NOOBS: New Out of the Box Software
PIR: passive IR

## References

[1] Niemeyer JE. Telemetry for small animal physiology. Lab Anim (NY). 2016;45(7): 255–7. 27327012 10.1038/laban.1048

[2] Johansen K, Krog J. Diurnal body temperature variations and hibernation in the birchmouse, Sicista betulina. Am J Physiol Content. 1959;196: 1200–1204. 10.1152/ajplegacy.1959.196.6.1200 13661341

[3] Cranford JA. Body temperature, heart rate and oxygen consumption of normothermic and heterothermic western jumping mice (Zapus princeps). Comp Biochem Physiol A Comp Physiol. 1983;74(3): 595–599. 6132704 10.1016/0300-9629(83)90553-4

[4] Waters JH, Stockley BH. Hibernating meadow jumping mouse on Nantucket Island, Massachusetts. J Mammal. 1965;46: 67–76.

[5] Larcombe A. Measurement of southern brown bandicoot (Isoodon obesulus) body temperature using internal and external telemeters. J R Soc West Aust. 2007;90: 161.

[6] McCauley MD, Wehrens XHT. Ambulatory ECG recording in mice. J Vis Exp JoVE. 2010; (39). pii: 1739. 10.3791/1739 PMC3152865 20517202

[7] Langer F, Fietz J. Ways to measure body temperature in the field. J Therm Biol. 2014;42: 46–51. 10.1016/j.jtherbio.2014.03.002 24802148

[8] Dausmann KH. Measuring body temperature in the field—evaluation of external vs. implanted transmitters in a small mammal. J Therm Biol. 2005;30: 195–202.

[9] Clarke RL, Smith RF, Justesen DR. An infrared device for detecting locomotor activity. Behav Res Methods, Instruments, Comput. 1985;17: 519–525.

[10] Siepka SM, Takahashi JS. Methods to record circadian rhythm wheel running activity in mice. Methods in enzymology. 2005;393: 230–239. 10.1016/S0076-6879(05)93008-5 15817291 PMC3770725

[11] Zurn JB, Jiang X, Motai Y. Video-based rodent activity measurement using near-infrared illumination. In: 2005 IEEE Instrumentation and Measurement Technology Conference Proceedings 2005. IEEE; 2005. pp. 1928–1931.

[12] Fowler SC, Birkestrand BR, Chen R, Moss SJ, Vorontsova E, Wang G, et al A force-plate actometer for quantitating rodent behaviors: illustrative data on locomotion, rotation, spatial patterning, stereotypies, and tremor. J Neurosci Methods. 2001;107: 107–124. 11389948 10.1016/s0165-0270(01)00359-4

[13] Jacky JP. A plethysmograph for long-term measurements of ventilation in unrestrained animals. J Appl Physiol. 1978;45: 644–647. 10.1152/jappl.1978.45.4.644 101497

[14] Stenina MA, Krivov LI, Spiridonov IN, Taranov AA. Remote Measurement of Respiratory Rate in Laboratory Mice during Studies of mdx-Model of Progressive Duchenne Muscular Dystrophy. Biomed Eng (NY). 2015;49: 79–84. 26477089

[15] Barbosa Pereira C, Kunczik J, Zieglowski L, Tolba R, Abdelrahman A, Zechner D, et al Remote Welfare Monitoring of Rodents Using Thermal Imaging. Sensors. 2018;18: 3653. 10.3390/s18113653 PMC6263688 30373282

[16] Cilulko J, Janiszewski P, Bogdaszewski M, Szczygielska E. Infrared thermal imaging in studies of wild animals. Eur J Wildl Res. 2013;59: 17–23.

[17] Pierce AJ, Pobprasert K. A portable system for continuous monitoring of bird nests using digital video recorders. J F Ornithol. 2007;78: 322–328.

[18] Chagas AM, Prieto-Godino LL, Arrenberg AB, Baden T. The€ 100 lab: A 3D-printable open-source platform for fluorescence microscopy, optogenetics, and accurate temperature control during behaviour of zebrafish, Drosophila, and Caenorhabditis elegans. PLoS Biol. 2017;15(7): e2002702 10.1371/journal.pbio.2002702 28719603 PMC5515398

[19] Cybulski JS, Clements J, Prakash M. Foldscope: origami-based paper microscope. PLoS ONE. 2014;9(6): e98781 10.1371/journal.pone.0098781 24940755 PMC4062392

[20] Whitesides GM. The frugal way. In: The Economist. 2011 Nov.

[21] Jordan B, Vercammen P, Cooper KL. Husbandry and breeding of the lesser Egyptian Jerboa, Jaculus jaculus. Cold Spring Harb Protoc. 2011;2011: 1457–1461. 10.1101/pdb.prot066712 22135654

[22] Jacobs GH. Evolution of colour vision in mammals. Philos Trans R Soc B Biol Sci. 2009;364: 2957–2967. 10.1098/rstb.2009.0039 19720656 PMC2781854

