# Supplementary material for: Nesting box imager: Contact-free, real-time measurement of activity, surface body temperature, and respiratory rate applied to hibernating mouse models
Source: PLoS Biol. 2019 Jul 24;17(7):e3000406. doi: 10.1371/journal.pbio.3000406 (PMC6682158; doi:10.1371/journal.pbio.3000406)
Supplement: S1 Text — PIR, passive infrared. (PDF) [file pbio.3000406.s001.pdf]

Recording Equipment

For validation of the NBI PIR, recordings were made with simultaneous data collection using commercial equipment from Sable Systems International. NBI PIR recordings were made using a modification the python code in the S1 Code, as provided in S3 Code. The purpose of this modification was to record the state of the PIR sensor (0 = not triggered, 1 = triggered) and date/time every 0.5 seconds; this change in data output was made to more easily allow integration of the NBI data with the Sable Systems data. Animal activity was recorded using a Sable Systems ADX-C Pad placed under the cage. Data was passed to Sable Systems ExpeData software by an ADX Reader and sampled every 2 seconds. Two additional PIR motion sensors were temporarily mounted to the underside of a standard cage lid, approximately 1/3 of the length of the lid from each end, to allow optimum coverage of the cage floor by the field of view of the sensors. These PIR sensors were powered by a 5V DC power source, and the output voltage of each was read into the ExpeData software via a Sable Systems UI-3 Universal Interface voltage channel and recorded with the same sample frequency as the ADX-C Pad. The voltage output of a light sensor was also sampled via the UI-3 to record the exact timing of the light/dark cycle.

Data Analysis

For comparison of Sable Systems and NBI data, the NBI data output text file was reformatted from UNIX to Windows format, the separator was changed to a comma, and the leading zeros from the dates were removed to allow import into the ExpeData software. The text processing was performed with the following set of awk commands, which can be run from an appropriate UNIX-like terminal.

```
awk 'sub("$", "\r")' Motion.txt | awk 'NR>3 { OFS=","; if($1 == "End") exit; gsub(/^0/,"",$1);
gsub(/\/0/,"/",$1);print $0}' > Motion_for_ExpeData.txt
```

Here, “Motion.txt” is the input file that is created by the modified NBI python script. The output filename is given as “Motion_for_ExpeData.txt” in this example. Either filename can be specified by the user. The result of this command is to drop any non-data lines of text and to convert data from this format:

```
05/10/2019 12:55:25 1
```

To this format, for ExpeData import:

```
5/10/2019,12:55:25,1
```

The NBI data were imported into the corresponding ExpeData recordings and automatically aligned by timestamp by the ExpeData software.

Briefly, working copies of the original data files were processed as follows: ADX data were processed to remove minor baseline noise, then the channel was zeroed to a period of no animal activity and any remaining data points below zero were clipped to zero. Raw motion data from the cage-top PIR sensor output voltage is approximately 0 V if no motion was detected, and approximately 3.3 V if the sensor detected motion. The raw PIR voltage data were standardized by clipping, such that any value below 0.5 V was set to 0, and any value greater than 1 V was set to 1. The two cage-top PIR channels were then summed to a new channel as ‘Cage-top PIR Motion.’ The ‘Total PIR Motion’ data were obtained by

adding the imported ‘NBI Atrium Motion’ data, which needed no further processing, to the ‘Cage-top PIR Motion’ data. This resulted in an equal weighting of each of the 3 PIR sensor outputs in the ‘Total PIR Motion’ data, as motion from each sensor could contribute one arbitrary unit of motion. To ease the workload of processing of multiple data files, the workflow was saved as a macro in ExpeData to allow batch processing of files (provided below).

For statistical analysis, 24-hour periods were selected using the light sensor data, starting with the beginning of the dark cycle, and the processed data channels from each 24-hour period were integrated over time. The integrated activity data were exported as a comma separated values (.csv) file and processed in Microsoft Excel to calculate the Pearson correlation coefficients (using the CORREL function) which are reported in S4 Table, and as summary statistics in S5 Table, below. The data were also normalized to a maximum value of unity and plotted to allow visual inspection as seen in S3 Fig, also provided below.

For actogram figure generation, 48-hour periods of the relevant channels, including light sensor data, were plotted using the ExpeData software. Given the limited graphical abilities of ExpeData (i.e., no capability to save plotted images), screenshots of the resulting plots were captured using MS Paint, open areas of the plots were filled with color when needed (e.g., gray color depicting light/dark cycle, open areas under the curve during high animal activity). Finally, the text, axis tick marks, and darkened baselines were added using Adobe Illustrator to improve figure readability.
