# Supplementary material for: Nesting box imager: Contact-free, real-time measurement of activity, surface body temperature, and respiratory rate applied to hibernating mouse models
Source: PLoS Biol. 2019 Jul 24;17(7):e3000406. doi: 10.1371/journal.pbio.3000406 (PMC6682158; doi:10.1371/journal.pbio.3000406)
Supplement: S2 Text — (PDF) [file pbio.3000406.s002.pdf]

To evaluate the fidelity of this image recognition-based algorithm, motion frequencies were measured for a model breathing system. To achieve a more controllable system for evaluation and troubleshooting, a model mouse was constructed by taping a blue nitrile glove to an orbital shaker. The orbital shaker was used to simulate translational motion associated with breathing. One end of the glove was attached to a stationary object to simulate the stretching and deformation also characteristic of breathing (S5 Fig).

Over 40 min, the orbital shaker was set to six different frequencies: 30, 60, 90, 120, 180, and 240 RPM. Three different regions of interest were used to evaluate repeatability of this algorithm. For each region, two frequencies were extracted based on motion along the X- and Y- axes. This produced a total of six responses for analysis (S6 Fig). Of these six responses, two exhibited erroneous responses at low frequencies. Low frequency error likely results from the inability of the median filter to remove sustained (slower motion = longer time) interference. Use of a higher-order median filter may mitigate this low-frequency error. Each region of interest, however, did return an accurate measurement across at least one axis. With actual mice, only one spatial axis is expected to produce usable data, as the mouse is not expected to exhibit similar breathing motions along both axes.
