# Supplementary material for: Nesting box imager: Contact-free, real-time measurement of activity, surface body temperature, and respiratory rate applied to hibernating mouse models
Source: PLoS Biol. 2019 Jul 24;17(7):e3000406. doi: 10.1371/journal.pbio.3000406 (PMC6682158; doi:10.1371/journal.pbio.3000406)
Supplement: S3 Text — (PDF) [file pbio.3000406.s003.pdf]

Running Video Analysis

First, the video from the Raspberry Pi camera must be converted from a .h264 extension to a .mp4 file. This may be achieved by executing Part 1 of MouseVideoTest. Execution requires the ffmpeg toolbox addon which may be accessed by the following link:

https://www.mathworks.com/matlabcentral/fileexchange/42296-ffmpeg-toolbox

Next, a feature from the video must be selected to track respiratory motion. This feature must be recognizably brighter or darker than its surroundings so that it is retained in a binary image. The binary image (an image with pixel values of "1" or "0") is constructed with the most or least intense fraction of pixels. Cutoff criteria is based on a user-defined threshold (variable name "Threshold", line 54) that is used in a comparison operation (line 57). This threshold is applied to the range of pixel intensities (defined as the difference between the brightest and darkest pixels) to determine a cutoff intensity (variable name "UpperCut"). Binary image data (variable name "BW") is obtained as the output of the comparison between image pixel values (variable name "I") and the cutoff:

```
BW = I>UpperCut;
```

If the "less than" operator is used, pixels that are darker than the cutoff will be retained as followable objects. If the "greater than" operator is used, brighter pixels will be retained.

Following selection of cutoff criteria, Part 2 of MouseVideoTest may be executed. After loading video data, window containing the first frame of the mouse video will appear. The user must then select a region of interest surrounding a feature that may be distinguished by the previously defined cutoff criteria. This region may be defined by writing coordinates directly into the code or by using the *ginput* function to determine region bounds by clicking on the image (default, S9 Fig).

After a substantial computation time (20-30 min for a 40 min video), final frequency data (along with intermediate data) will be output. For a single followed feature, two responses will be plotted: one for x-axis motion and another for y-axis motion. It is common for only one of these two responses to correspond to breathing rate. The relevant response may be determined based on initial video footage.

Noise Troubleshooting

In cases where selection of an optimal region of interest is not sufficient or not feasible, the size of the time window (variable name "Window") used in Fourier transform analysis may be increased. If the output bounces between outliers, these values may skew the reported respiratory frequency. Relative to a defined frequency range of interest, these artifacts can positively bias low frequencies and negatively bias high frequencies.

While smoothing filters may be used to reduce noise, changing the size of the time window can have a more profound effect. Increasing sample size decreases the probability of misidentifying an outlier frequency peak as respiratory motion. The produces more stable and accurate frequency data, as shown in S8 Fig. The limitation, however, imposed by using wider time windows is poorer time resolution. For example, at the longest time window, this improved signal stabilization and accuracy is at the cost of missing a perturbation at approximately 660 s. Frequency plots shown in this paper were prepared using time windows of 66 s (2000 timepoints).
