# Supplementary figures and images for: Nesting box imager: Contact-free, real-time measurement of activity, surface body temperature, and respiratory rate applied to hibernating mouse models
Source: PLoS Biol. 2019 Jul 24;17(7):e3000406. doi: 10.1371/journal.pbio.3000406 (PMC6682158; doi:10.1371/journal.pbio.3000406)

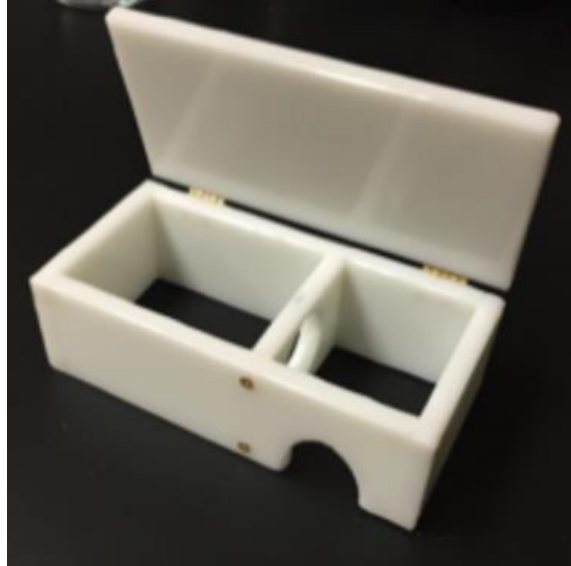

Supplement: S1 Fig — HDPE, high-density polyethylene. (PDF) [file pbio.3000406.s004.pdf]

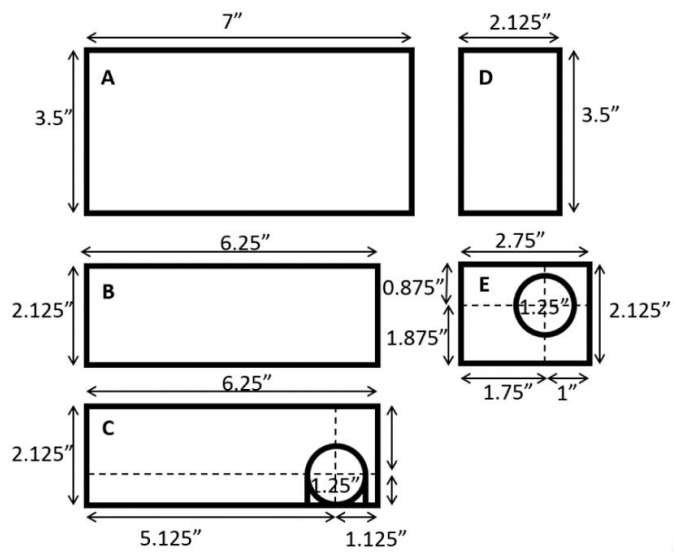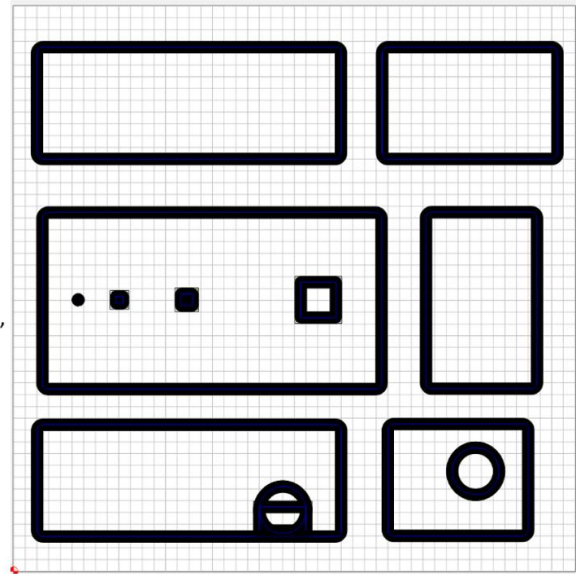

Supplement: S2 Fig — A = top plate; B = side plate (no hole); C = side plate, entrance hole; D = side walls (×2); and D = interior wall between atrium and nesting room. (Left) Cut out image from Shapeoko Carbide cutter, on 1/4-inch grid spacing. Additional cuts are made into part A to embed sensors. HDPE, high-density polyethylene. (PDF) [file pbio.3000406.s005.pdf]

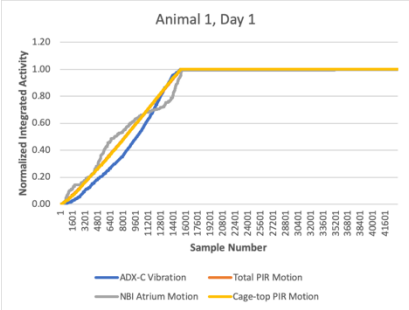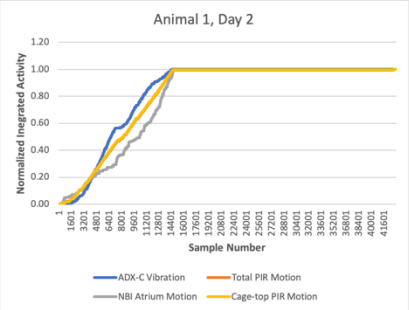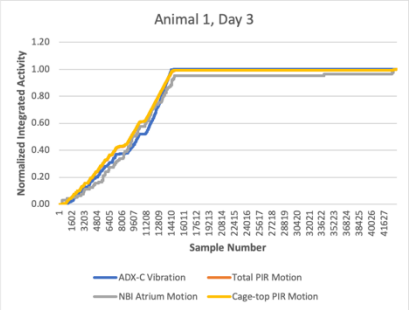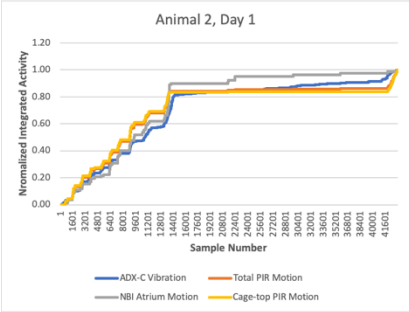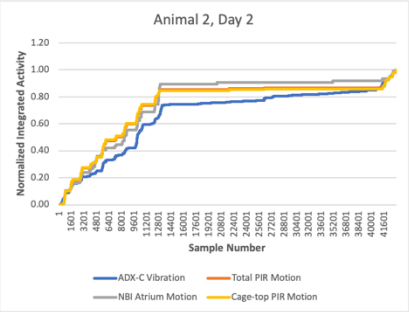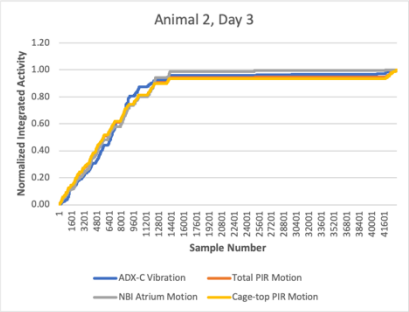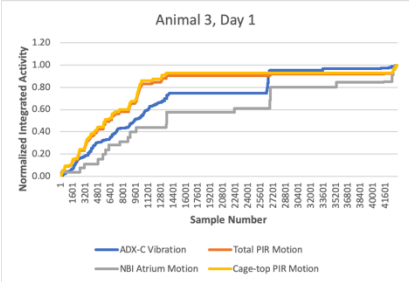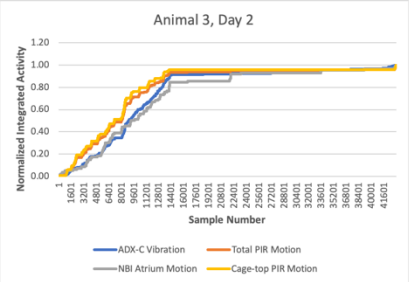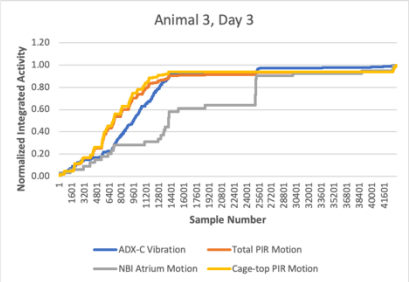

Supplement: S3 Fig — Each panel depicts 24 hours of motion starting with onset of dark cycle (n = 9 days, 3 days each from three different animals). Individual lines represent activity as recorded via ADX-C Vibration, NBI Atrium Motion, Cage-top PIR Motion, and Total PIR Motion, which is the sum of the Cage-top PIR Motion and NBI Atrium Motion. Individual animals have different patterns of activity, and the PIR-based motion sensor data generally correspond well with the commercial ADX-C vibration sensing pad. The data underlying this figure can be found in S4 Data. NBI, nesting box imager; PIR, passive infrared. (PDF) [file pbio.3000406.s006.pdf]

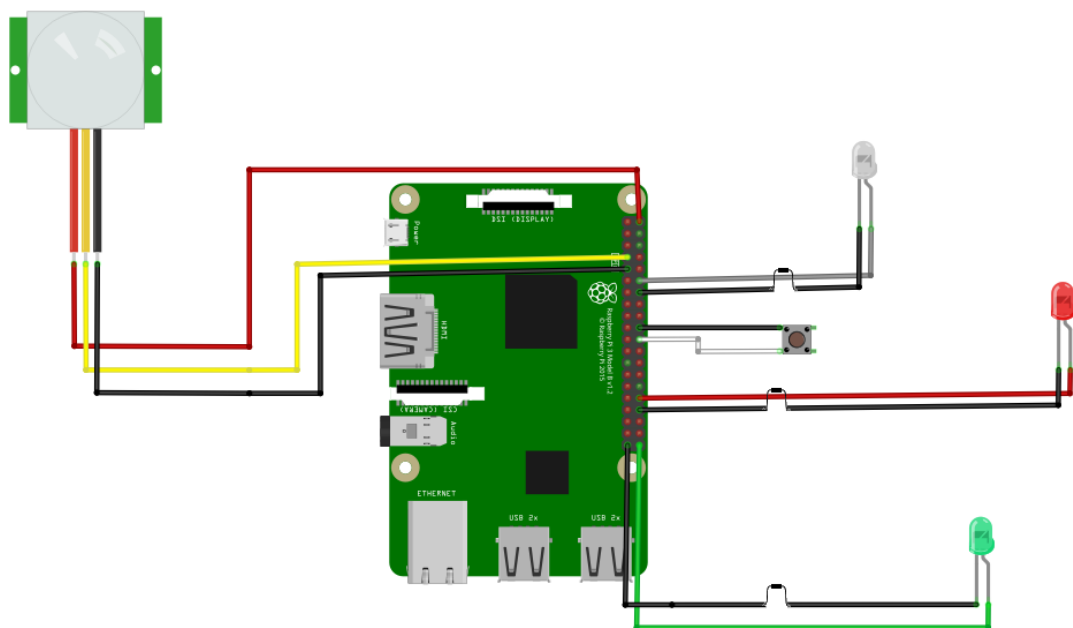

Supplement: S4 Fig — LEDs were connected in series with 220-Ω resistors to limit current. LED, light-emitting diode. (PDF) [file pbio.3000406.s007.pdf]

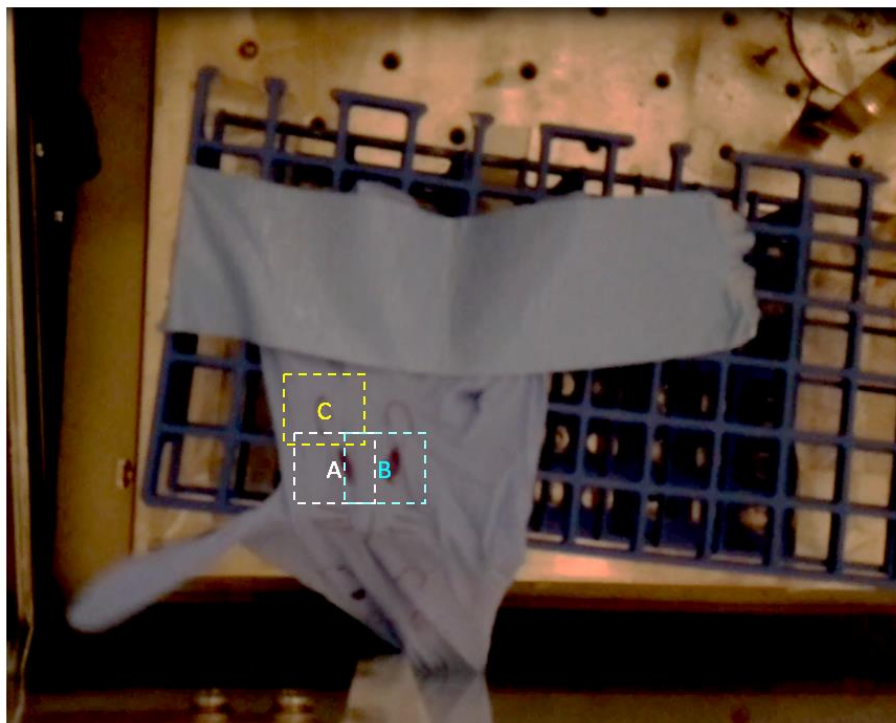

Supplement: S5 Fig — Dashed boxes enclose three different regions used in image analysis. (PDF) [file pbio.3000406.s008.pdf]

(a)

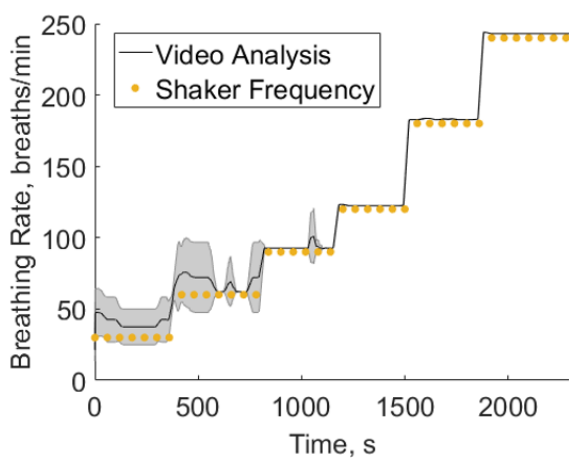

(b)

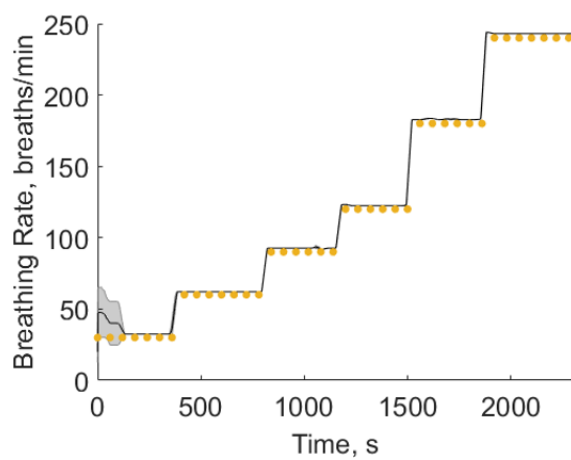

Supplement: S6 Fig — Respiratory rate data collected by analyzing mouse model using all X- and Y-motion data from each region (a) (n = 6) and using motion data after removing outliers (b) (n = 4). Shaded regions indicate a single standard deviation. The data underlying this figure can be found in S5 Data. (PDF) [file pbio.3000406.s009.pdf]

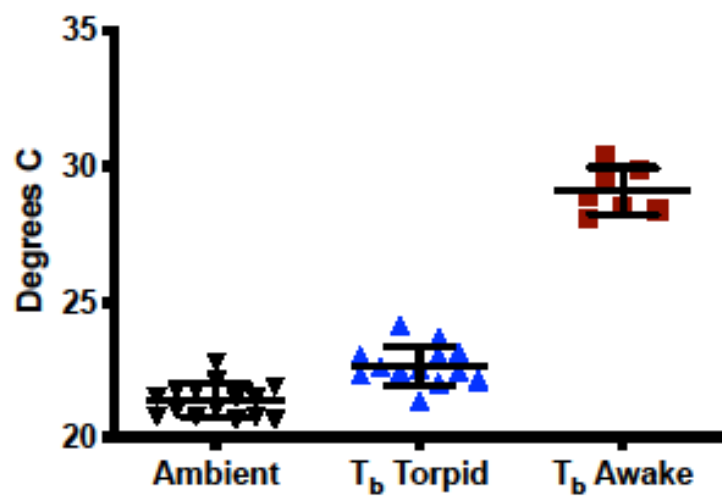

Supplement: S7 Fig — Surface temperatures were determined by periodic measurements of a group of seven mice over 23 days using an IR thermometer. Ambient temperatures were determined as the temperature of the bedding at the same time that the mice were measured (not all paired). Each mouse and bedding was only measured once on the day that they were measured. Error bars indicate single standard deviation calculated for 15, 13, and seven measurements for ambient, torpid, and awake temperatures, respectively. The data underlying this figure can be found in S6 Data. IR, infrared. (PDF) [file pbio.3000406.s010.pdf]

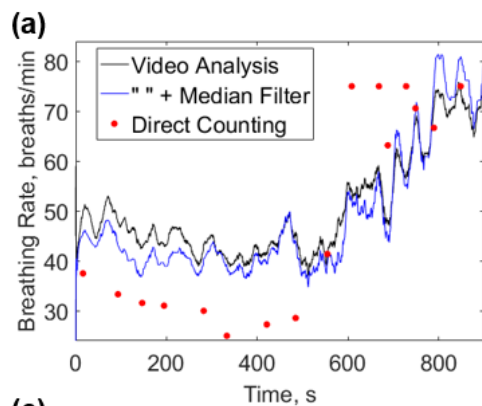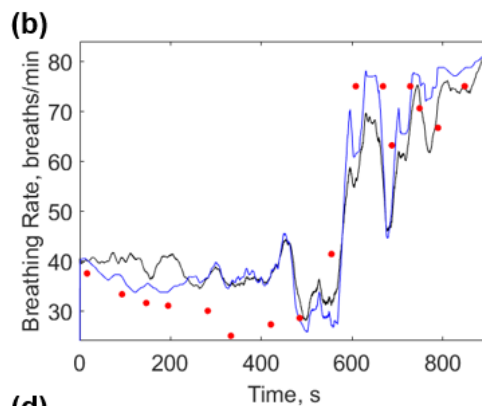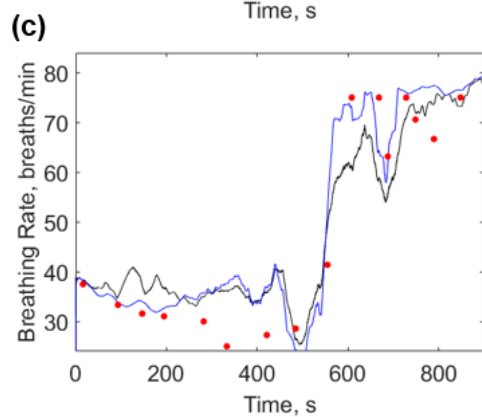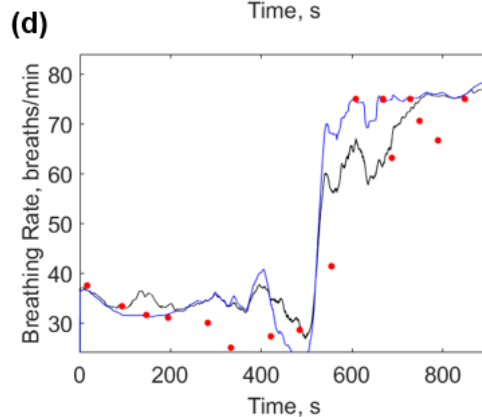

Supplement: S8 Fig — Different plots indicate algorithm outputs with timeframes (size of time window used in Fourier transform) of 13 seconds (a), 26 seconds (b), 40 seconds (c), and 66 seconds (d). Solid black lines indicate algorithm output using only a rolling average smoothing filter (average over 800 timepoints, 26 seconds). Solid blue lines are smoothed with a median filter prior to rolling average smoothing. Red dots are measurements collected by directly observing mouse footage. The data underlying this figure can be found in S3 Data. (PDF) [file pbio.3000406.s011.pdf]

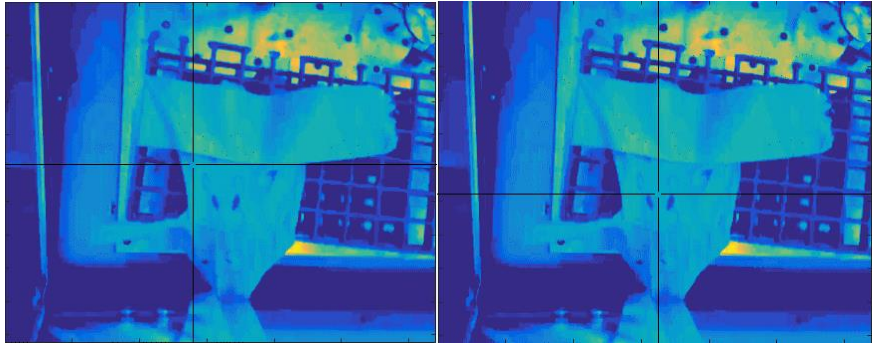

Supplement: S9 Fig — (Left) Upper-left bound of region C (shown in S5 Fig—“mouse” left ear), selected first. (Right) Lower-right bound of region C, selected second. (PDF) [file pbio.3000406.s012.pdf]
