# Supplementary material for: Nesting box imager: Contact-free, real-time measurement of activity, surface body temperature, and respiratory rate applied to hibernating mouse models
Source: PLoS Biol. 2019 Jul 24;17(7):e3000406. doi: 10.1371/journal.pbio.3000406 (PMC6682158; doi:10.1371/journal.pbio.3000406)
Supplement: S1 Table — (PDF) [file pbio.3000406.s013.pdf]

Measurement	Tool	Advantages	Limitations
Activity	IR motion detector	Direct detection of motion	Requires unobstructed view of subject, may not capture small movements (e.g., grooming, shivering).
	Force Plate Actometer	Direct measurement of animal locomotion	Requires direct contact of animal on plate
	Running Wheel	Direct measurement of animal running on wheel	Only detects wheel-running behavior; will not work for species with incompatible gait
	Vibration sensing (piezo under-cage pad)	Very sensitive and can detect small motions (e.g., shivering, grooming behavior)	Very sensitive to external vibration; cannot be employed in high-vibration environments such as environmental chambers
	IR Beam-Breaking	Reliable detection of gross motor activity. Sophisticated systems can track x-y-z motion of animal in cage	Obstructions in cage can prevent transmission of IR beam; systems are usually designed for empty cage.
Temperature	Thermocouple or Thermistor Probe (commonly oral, rectal, or body surface)	Accurate, direct measurement of temperature at probe	Requires restraint of subject, attachment, or possibly surgery to implant lead.
	Implantable Telemeter, Logger, or RFID Chip (commonly implanted subcutaneously or intraperitoneally)	Provides measurement of core T_b or subcutaneous temperature of free-moving subject	Requires surgery or subcutaneous injection; data acquisition may depend on nearby equipment or manual reading of RFID chip
	Hand-held IR Thermometer	Simple, direct measurement of surface temperature. Possible to collect data without restraining the subject. Relatively inexpensive.	Requires manual data collection, which may disturb subject depending on application.
	Thermal Camera	Provides surface temperature with spatial resolution. Can image unrestrained animals.	Commercial systems are expensive; cost prohibitive to scale to studies requiring parallel monitoring; may require manual data collection

Respiratory Rate	Video Analysis	Non-contact, Low sensor cost	Unable to detect rapid changes, High noise, most methods require a stationary or immobilized subject
	Plethysmography	Direct detection of respiration via air pressure changes.	Usually requires animal to be placed in sealed plethysmography chamber. Not easily integrated with long-term recording.
