# Supplementary material for: Nesting box imager: Contact-free, real-time measurement of activity, surface body temperature, and respiratory rate applied to hibernating mouse models
Source: PLoS Biol. 2019 Jul 24;17(7):e3000406. doi: 10.1371/journal.pbio.3000406 (PMC6682158; doi:10.1371/journal.pbio.3000406)
Supplement: S2 Table — (PDF) [file pbio.3000406.s014.pdf]

Product	order	Content	#	$	Site
Raspberry pi 3	1	Paspberry Pi 3 Model B	1	$35.00	https://www.element14.com/community/community/raspberry-pi
	2	Pi Cases	1	$6.99	https://www.element14.com/community/community/raspberry-pi/raspberry-pi-accessories?ICID=menubar_topics_rpiaccessories
	3	Cables and Power	1	$9.97	https://www.element14.com/community/community/raspberry-pi/raspberry-pi-accessories?ICID=menubar_topics_rpiaccessories
	4	MicroSD Card (Sandisk NOOBS Raspberry pi)	1	$13.99	https://www.element14.com/community/community/raspberry-pi/raspberry-pi-accessories?ICID=menubar_topics_rpiaccessories
	5	Female Female Jumper Wires (20 packs) (we need atleast 12)	1	$1.95	https://www.sparkfun.com/products/12796
	6	Keyboard & Mouse	1	$11.88	http://www.newegg.com/Product/Product.aspx?Item=9SIA67038W0518&cm_re=keyboard_and_mouse-_-9SIA67038W0518-_-Product
	7	HDMI Cable	1	$1.49	http://www.newegg.com/Product/Product.aspx?Item=N82E16882189028
	8	Monitor with HDMI port	1	$59.99	http://www.newegg.com/Product/Product.aspx?Item=N82E16824112027&cm_re=hdmi_monitor-_-24-112-027-_-Product
	9	USB (8GB)		$4.95	http://www.newegg.com/Product/Product.aspx?Item=N82E16820242067&cm_re=usb_8gb-_-20-242-067-_-Product
	-	Total Cost	-	$146.21	-
Mouse House	1	HDPE sheet 3/8" thickness 12" * 12" w*l	1	$13.31	https://www.amazon.com/Density-Polyethylene-Plastic-Sheet-Natural/dp/B017N0NFRE/ref=pd_lpo_328_lp_img_4?_encoding=UTF8&psc=1&refRID=ESJ2FA8KS942ZX84FT91
	2	Devcon 5 minute Epoxy	1	$8.49	https://www.grainger.com/product/DEVCON-Epoxy-5A462
	-	Total Cost	-	$21.80	-
Noir Camera	1	PI NOIR CAMERA V2	1	$25.00	https://www.element14.com/community/community/raspberry-pi/raspberry-pi-accessories/pinoir?ICID=rpiaccsy-access-products
	2	Ribbon Cable	1		(comes with pi noir camera V2)
	3	220 ohm resistor (5packs) (we need only 1)($1.49)	1	$0.30	https://www.radioshack.com/products/radioshack-220-ohm-1-4w-5-carbon-film-resistor-pk-5
	4	Infrared LED 890nm	1	$0.95	https://www.sparkfun.com/search/results?term=infrared+led
	-	Total Cost	-	$26.25	-
Thermal Camera	1	Flir Lepton Thermal Camera	1	$259.95	https://www.sparkfun.com/products/13233
	-	Total Cost	-	$259.95	-
Motion Sensor	1	PIR motion sensor	1	$9.95	https://www.sparkfun.com/products/13285
	2	Female Male Cable (20 packs) (we need at least 3)	1	$1.95	https://www.sparkfun.com/products/12794
	-	Total Cost	-	$1.95	-
