# Supplementary material for: Nesting box imager: Contact-free, real-time measurement of activity, surface body temperature, and respiratory rate applied to hibernating mouse models
Source: PLoS Biol. 2019 Jul 24;17(7):e3000406. doi: 10.1371/journal.pbio.3000406 (PMC6682158; doi:10.1371/journal.pbio.3000406)
Supplement: S3 Table — The red highlighted boxes connect to the thermal camera. The orange highlighted boxes connect to the PIR motion sensor. The gray, light-red, and green highlighted boxes connect to the NIR, red, and green LEDs, respectively. The blue highlighted boxes connect to the safe shutoff button. GPIO, general-purpose input/output; LED, light-emitting diode; NIR, nesting box imager; PIR, passive infrared. (PDF) [file pbio.3000406.s015.pdf]

Wire	Name	P1 Pin Number		Name	Wire
VIN	3.3v DC Power	1	2	5v DC Power	Left(1) (Red)(+)
SDA	GPIO02 (SDA1, I2C)	3	4	5v DC Power	
SCL	GPIO03 (SCL1, I2C)	5	6	Ground	GND
Middle(2) (White)	GPIO04 (GPIO_GCLK)	7	8	GPIO14 (TXD0)	
Right(3) (Black)(AL)	Ground	9	10	GPIO15 (RXD0)	
	GPIO17 (GPIO_GEN0)	11	12	GPIO18 (GPIO_GEN1)	NIR LED Anode
	GPIO27 (GPIO_GEN2)	13	14	Ground	NIR LED Cathode
	GPIO22 (GPIO_GEN3)	15	16	GPIO23 (GPIO_GEN4)	
	3.3v DC Power	17	18	GPIO24 (GPIO_GEN5)	
MOSI	GPIO10 (SPI_MOSI)	19	20	Ground	Power Button
MISO	GPIO09 (SPI_MISO)	21	22	GPIO25 (GPIO_GEN6)	Power Button
CLK	GPIO11 (SPI_CLK)	23	24	GPIO08 (SPI_CE0_N)	CS
	Ground	25	26	GPIO07 (SPI_CE1_N)	
	ID_SD (I2C ID EEPROM)	27	28	ID_SC (I2C ID EEPROM)	
	GPIO05	29	30	Ground	
	GPIO06	31	32	GPIO12	Red LED Anode
	GPIO13	33	34	Ground	Red LED Cathode
	GPIO19	35	36	GPIO16	
	GPIO26	37	38	GPIO20	
Green LED Anode	Ground	39	40	GPIO21	Green LED Cathode
