# Supplementary material for: Nesting box imager: Contact-free, real-time measurement of activity, surface body temperature, and respiratory rate applied to hibernating mouse models
Source: PLoS Biol. 2019 Jul 24;17(7):e3000406. doi: 10.1371/journal.pbio.3000406 (PMC6682158; doi:10.1371/journal.pbio.3000406)
Supplement: S4 Table — PIR, passive infrared. (PDF) [file pbio.3000406.s016.pdf]

Animal	1			2			3		
Day	1	2	3	1	2	3	1	2	3
**ADX-C : Total PIR Motion**	0.997	0.993	0.997	0.984	0.979	0.997	0.933	0.989	0.976
**ADX-C : NBI Atrium Motion**	0.986	0.967	0.996	0.997	0.987	0.995	0.986	0.995	0.907
**ADX-C : Cage-Top PIR Motion**	0.997	0.993	0.997	0.977	0.976	0.997	0.920	0.982	0.966
