# Supplementary material for: Nesting box imager: Contact-free, real-time measurement of activity, surface body temperature, and respiratory rate applied to hibernating mouse models
Source: PLoS Biol. 2019 Jul 24;17(7):e3000406. doi: 10.1371/journal.pbio.3000406 (PMC6682158; doi:10.1371/journal.pbio.3000406)
Supplement: S5 Table — PIR, passive infrared. (PDF) [file pbio.3000406.s017.pdf]

	Mean	Min	Max
**ADX-C : Total PIR Motion**	0.977	0.933	0.997
**ADX-C : NBI Atrium Motion**	0.978	0.907	0.997
**ADX-C : Cage-Top PIR Motion**	0.970	0.920	0.997
