# Supplementary material for: Nesting box imager: Contact-free, real-time measurement of activity, surface body temperature, and respiratory rate applied to hibernating mouse models
Source: PLoS Biol. 2019 Jul 24;17(7):e3000406. doi: 10.1371/journal.pbio.3000406 (PMC6682158; doi:10.1371/journal.pbio.3000406)
Supplement: S6 Table — NOOBS, New Out of the Box Software. (PDF) [file pbio.3000406.s018.pdf]

Library	Source
opencv	Python Package Index
pylepton	https://github.com/kekiefer/pylepton.git
ffmpeg	Python Package Index
