# Supplementary material for: Nesting box imager: Contact-free, real-time measurement of activity, surface body temperature, and respiratory rate applied to hibernating mouse models
Source: PLoS Biol. 2019 Jul 24;17(7):e3000406. doi: 10.1371/journal.pbio.3000406 (PMC6682158; doi:10.1371/journal.pbio.3000406)
Supplement: S1 Code — (PDF) [file pbio.3000406.s019.pdf]

Python Code for Thermal Camera

```
# To Activate Thermal Camera
import numpy as np
import cv2

#To Activate sleep(9) function
from pylepton import Lepton
import time
from datetime import datetime

# To Active LED
import RPi.GPIO as GPIO
GPIO.setmode(GPIO.BCM)
GPIO.setwarnings(False)
GPIO.setup(21,GPIO.OUT)

GPIO.output(21,1) # LED on

while True:
    filename0 = datetime.now().strftime("%Y%m%d%H%M%S\n")
    print("Start")
    print(filename0)

    #Thermo Camera
    filename2 =
datetime.now().strftime("/media/pi/DATA/mousehouse/thermodata/Raw%Y%m%
d%H%M%S.txt")
    filename4 =
datetime.now().strftime("/media/pi/DATA/mousehouse/thermalimage/thermo
%Y%m%d%H%M%S.jpg")
    with Lepton() as l:
      a,_ = l.capture()
    np.savetxt(filename2,a)
    cv2.normalize(a, a, 0, 67070, cv2.NORM_MINMAX) # extend contrast
    np.right_shift(a, 8, a) # fit data into 8 bits
    cv2.imwrite(filename4, np.uint8(a)) # write it!

    filename10 = datetime.now().strftime("%Y%m%d%H%M%S\n")
    print("end")
    print(filename10)

    # Sleep function- record every 10 seconds
    #time.sleep(9)
```

Python Code for Motion Sensor

```
from gpiozero import MotionSensor
from datetime import datetime

pir = MotionSensor(4)
```

```
# Added by NFR on 1.3.2018, we need to record the START time of the
experiment
Txt = open("/media/pi/DATA/mousehouse/Motion.txt",'a')
Txt.write("Start Time:\n")
filename = datetime.now().strftime("%Y%m%d%H%M%S\n")
#print(filename)
Txt.write(filename)
Txt.write("Start Motion measures:\n")
while True:
    if pir.motion_detected:
        #Txt = open("/media/pi/DATA/mousehouse/Motion.txt",'a')
        filename = datetime.now().strftime("%Y%m%d%H%M%S\n")
        #print(filename)
        Txt.write(filename)
```

Python Code for Button and Motion Sensor

```
import time
import os

# To Active NIR LED
import RPi.GPIO as GPIO
GPIO.setmode(GPIO.BCM)
GPIO.setwarnings(False)
GPIO.setup(18,GPIO.OUT)

from picamera import PiCamera
from time import sleep
from datetime import datetime
camera = PiCamera()

#Button trigger
import RPi.GPIO as GPIO
GPIO.setmode(GPIO.BCM)
GPIO.setup(25, GPIO.IN, pull_up_down=GPIO.PUD_UP)

# To Active LED
import RPi.GPIO as GPIO
GPIO.setmode(GPIO.BCM)
GPIO.setwarnings(False)
GPIO.setup(12,GPIO.OUT)

GPIO.output(18,1) # LED on
time.sleep(0.1)
filename0 =
datetime.now().strftime("/media/pi/DATA/mousehouse/noirimage/%Y%m%d%H%
M%S.h264")
camera.start_recording(filename0)
while True:
    print ("hi")
    input_state = GPIO.input(25)
    if input_state == False:
```

```
        print ("poweroff")
        camera.stop_recording()
        # Added 1.3.2018 by NFR Also record end time to experiment in
motion file
        Txt = open("/media/pi/DATA/mousehouse/Motion.txt",'a')
        Txt.write("End Time:\n")
        filename = datetime.now().strftime("%Y%m%d%H%M%S\n")
        Txt.write(filename)
        Txt.write("End Experiment\n")
        Txt.close()
        GPIO.output(12,0)
        time.sleep(0.5)
        GPIO.output(12,1)
        time.sleep(0.5)
        GPIO.output(12,0)
        time.sleep(0.5)
        GPIO.output(12,1)
        time.sleep(0.5)
        GPIO.output(12,0)
        time.sleep(0.5)
        GPIO.output(12,1)
        time.sleep(1)
        os.system("poweroff")
    time.sleep(0.2)
```

Python code used to capture motion data for processing in ExpeData software.

```
# Coded By Han Jong Shin, modified by WJI

from gpiozero import MotionSensor
from datetime import datetime
import time

#Button trigger; added this and writing of begin & end times 1.4.2018;
WJI, note now have to hold button for longer than 0.5 sec
import RPi.GPIO as GPIO
GPIO.setmode(GPIO.BCM)
GPIO.setup(25, GPIO.IN, pull_up_down=GPIO.PUD_UP)

Txt = open("/media/pi/DATA/mousehouse/Motion.txt",'a')
Txt.write("Begin Time:\n")
filename = datetime.now().strftime("%m/%d/%Y %H:%M:%S\n")
Txt.write(filename)
Txt.write("Continue Experiment\n")
Txt.close()

pir = MotionSensor(4)
while True:
    input_state = GPIO.input(25)
    if input_state == False:
        Txt = open("/media/pi/DATA/mousehouse/Motion.txt",'a')
        Txt.write("End Time:\n")
```

```
        filename = datetime.now().strftime("%m/%d/%Y %H:%M:%S\n")
        Txt.write(filename)
        Txt.write("End Experiment\n")
        Txt.write("----------\n")
        Txt.close()
        time.sleep(0.5)
        quit()
    elif pir.motion_detected:
        Txt = open("/media/pi/DATA/mousehouse/Motion.txt",'a')
        filename = datetime.now().strftime("%m/%d/%Y %H:%M:%S 1\n") #1
= motion, dates formatted for ExpeData import after processing
        print(filename)
        #print(input_state)
        Txt.write(filename)
        Txt.close()
        time.sleep(0.5)
    else:  #added 2019-04-05 for motion sensor recording, prints when
no motion detected
        Txt = open("/media/pi/DATA/mousehouse/Motion.txt",'a')
        filename = datetime.now().strftime("%m/%d/%Y %H:%M:%S 0\n") #
0 = no motion
        print(filename)
        Txt.write(filename)
        Txt.close()
        time.sleep(0.5)
```
