# Supplementary material for: Nesting box imager: Contact-free, real-time measurement of activity, surface body temperature, and respiratory rate applied to hibernating mouse models
Source: PLoS Biol. 2019 Jul 24;17(7):e3000406. doi: 10.1371/journal.pbio.3000406 (PMC6682158; doi:10.1371/journal.pbio.3000406)
Supplement: S2 Code — (PDF) [file pbio.3000406.s020.pdf]

```
function MouseMove
% Coded on 8.24.2018 by NFR
D = xlsread('comboruns_clean.xlsx');
% This data is all of the motion data without the header files.
% First row is the experiment start time
% Last row is the experiment end time
% Data structure is YYYYMMDDHHMMss
% Convert to string to peel off the hours, minutes and seconds....this
% works for data that is collected on a single day.
S = num2str(D);
H = str2num(S(:,9:10));
M = str2num(S(:,11:12));
s = str2num(S(:,13:14));
% Now we need to convert to time elapsed vector (in seconds)
TEV = zeros(length(H),1);
for i = 1:length(H)
    time = (H(i,1)-H(1,1))*3600 + (M(i,1)-M(1,1))*60 + (s(i,1)-s(1,1));
    TEV(i,1) = time;
end
% Now plot the activity
N = ones(length(TEV),1);
plot(TEV./60,N,'o')
xlabel('Time (min)')
csvwrite('TEV.csv',TEV);

end

function ThermoVec
% Coded by NFR on 8.24.18
% This code converts the thermo files to a total intenstity vs. time stamp
%
% Read all file names
fileList = dir('*.txt');
n = {fileList.name};
%What is the start HH, MM, SS
str1 = n{1};
sH = str2double(str1(12:13));
sM = str2double(str1(14:15));
sS = str2double(str1(16:17));
% Create vector for time and intensity
Data = zeros(length(n)-1,2);
for i = 1:length(n)-1
    % Read in file name to get time stamp
    str1 = n{i};
    tH = str2double(str1(12:13));
    tM = str2double(str1(14:15));
    tS = str2double(str1(16:17));
    % Record time elapsed
    Data(i,1) = (tH-sH)*3600+(tM-sM)*60 + (tS-sS);
    Idata = dlmread(n{i});
    Isort = sort(Idata(:),'descend');
    %4800 total pixels, let's look at top 10%
    Data(i,2) = mean(Isort(1:4800*.20));
    m = 10;

end
plot(Data(:,1),Data(:,2))
csvwrite('ThermoData_2.csv',Data)
```

```
end

function plotTherm_v2
% Coded by NFR on 8.25.18
% This code plots the temp change of mouse on Jan 30, 2018
% This is the same mouse that Nathaniel has shown increased breathing rate
% v2 looks at data from the SECOND video ONLY, also uses calibration to
% plot as temperature!
%
D = csvread('ThermoData_2.csv');
t = 0;
I = 0;
count = 1;
for i = 1:length(D)
    if D(i,2) < 9200
        t(count,1) = D(i,1)-1200;
        I(count,1) = D(i,2);
        count = count +1;
    end
end
%plot(t,I,'.')
%ylabel('Lepton Units')
%xlabel('Time (s)')
%
% Let's plot in actual temperature units!
CD = csvread('CalData.csv');
Temp = spline(CD(:,2),CD(:,1),I);
plot(t,Temp,'.')
ylabel('Temp (°C)')
xlabel('Time (s)')
xlim([0 1400])
ylim([20 26])
end
function MouseVideoTest
% Coded by NEK and NFR on 4.26.2018
% Video Conversion Coded by NFR and HJS on 11.15.2017

% V3 - attempted 'smart' process to ensure that the centroid is NOT jumping
% around frame to frame
%
% w = 1920, H = 1080, Frame rate = 30, BPP = 24


% Step 1 - Convert video to MP4
%{
ffmpegDir = 'C:\ffmpeg-20171114-91a565e-win64-static';
cmd = ['"' fullfile(ffmpegDir, 'bin', 'ffmpeg.exe') '" -r 30 -i video2.h264 -
vcodec copy Cvideo2.mp4 &'];
[status, message] = system(cmd);
%}

% Step 2 - Convert video to frames

 vid=VideoReader('breathingcalibration.mp4');
 numFrames = vid.NumberOfFrames;
 n=numFrames;
```

```
%Step 3 - Find the region of interest
 % 11.29.2017 Use Green Channel
 figure(1)
 clf
frame = read(vid,1); % Data in unsigned integer
Green_Data = double(frame(:,:,2));
imagesc(Green_Data);
[nr,nc] = ginput(2);

%MaxGreenCount = max(Green_Data(:))
%MinGreenCount = min(Green_Data(:))
%
%Fraction of video being analyzed
m = round(n/1);
%
ROIpic = Green_Data(round(nc(1)):round(nc(2)),round(nr(1)):round(nr(2)));
ROIvec = zeros(3,2,m); % First column is x data fo first ROI and second
column is y data
sROIvec = zeros(3,2,m);

%
% Added on 4.9.2018 by NFR to check and improve the centroid finding routine
%v = VideoWriter('CentroidVideo_v4.avi');
%open(v)
%

for i = 1:m
   frame = read(vid,i); % Data in unsigned integer
   Green_Data = double(frame(:,:,2));
   I = Green_Data(round(nc(1)):round(nc(2)),round(nr(1)):round(nr(2)));
   Imax = max(I(:));
   Imin = min(I(:));
   Threshold = .80; % upper percentage of pixels that are removed to display
as 0s
   Span = (Imax-Imin);
   UpperCut = round(Imax-(Span)*Threshold);
   BW = I<UpperCut;
   %[labeled,numObjects] = bwlabel(bw,4);% Label components.
   %graindata = regionprops(labeled,'basic')
   %graindata(51).Area
   s = regionprops(BW,'centroid','area');
   centroids = cat(1, s.Centroid);
   areas = cat(1, s.Area);
   combo = [centroids areas];
   CS = flipud(sortrows(combo,3));

   if i < 300
   % Added by NFR on 4.9.2018
   imagesc(BW)
   hold on
   ncp = 1;
   plot(CS(1:ncp,1),CS(1:ncp,2),'g.')
   for k = 1:ncp
       t = text(CS(k,1),CS(k,2),int2str(k));
       t.Color = 'r';
   end
```

```
    hold off
    % Record image as frame
    %frame = getframe(gcf);
    %writeVideo(v,frame);
    end

    %ROIvec(:,:,i) = CS(1:3,1:2);  % 4.21 I think there was an error here in
v2, it was recording the area column, not the XY coordinates
    % Let's put in some 'smartness' to the algorithm here to determine if
    % the area of the centroid is not changing between frames...if it is,
    % it is likely jumping to some other centroid
    %
    % Centroid check
    if ~(i == 1)
        for k = 1:1
            if abs(CS(k,3)-CS_old(k,3)) <= 80 % you may need to adjust this
tolerance
                % Record the xy location of the centroid;
                ROIvec(k,:,i) = CS(k,1:2);
            else
                ROIvec(k,:,i) = CS_old(k,1:2);
            end
        end
    end
    CS_old = CS;
end
%close(v)
figure(2)
clf
timevec = ((1:m)./30)';
% 4.21.18 Create new vector that removes the zero points
ROI1 = [0 0 0];
ROI2 = [0 0 0];
ROI3 = [0 0 0];
c1 = 1;
c2 = 2;
c3 = 3;
for i = 1:m
    if ROIvec(1,1,i) > 0
        ROI1(c1,:) = [timevec(i),ROIvec(1,1,i),ROIvec(1,2,i)];
        c1 = c1 + 1;
    end
    if ROIvec(2,1,i) > 0
        ROI2(c2,:) = [timevec(i),ROIvec(2,1,i),ROIvec(2,2,i)];
        c2 = c2 + 1;
    end
    if ROIvec(3,1,i) > 0
        ROI3(c3,:) = [timevec(i),ROIvec(3,1,i),ROIvec(3,2,i)];
        c3 = c3 + 1;
    end
end

% First ROI
subplot(3,2,1)
plot(ROI1(:,1),ROI1(:,2))
xlabel('Time (s)')
```

```
ylabel('X position')
subplot(3,2,2)
plot(ROI1(:,1),ROI1(:,3))
xlabel('Time (s)')
ylabel('Y position')
% Second ROI
subplot(3,2,3)
plot(ROI2(:,1),ROI2(:,2))
xlabel('Time (s)')
ylabel('X position')
subplot(3,2,4)
plot(ROI2(:,1),ROI2(:,3))
xlabel('Time (s)')
ylabel('Y position')
% Third ROI
subplot(3,2,5)
plot(ROI3(:,1),ROI3(:,2))
xlabel('Time (s)')
ylabel('X position')
subplot(3,2,6)
plot(ROI3(:,1),ROI3(:,3))
xlabel('Time (s)')
ylabel('Y position')

for ii = 1:m
    XPos(:,ii) = ROIvec(:,1,ii);
    YPos(:,ii) = ROIvec(:,2,ii);
end

plot(timevec,XPos);
plot(timevec,YPos);

%Initialize Figures
figure(5)
clf
figure(6)
clf
figure(55)
clf

%Step 4 - Analyze position data
%Plot the Fourier transform of the data set collected from the entire video
figure(4)
%Convert from time to frequency domain
FreqSpace(:,1) = imag(fft(XPos(1,:)));
FreqSpace(:,2) = imag(fft(YPos(1,:)));
plot((1:length(FreqSpace((1:(length(FreqSpace)/2)),1)))/(length(FreqSpace(:,1
)))*30,FreqSpace(1:(length(FreqSpace(:,1))/2),1).^2);
xlim([0 7.5]);
xlabel('Frequency, Hz')
ylabel('Intensity')


%Each index value of the time array corresponds to 1/30 of a second
%When converted to an fft array, a symmetrical array is produced with a
%number of elements equal to the number of timepoints.
```

```
%The first half of the transformed array corresponds to the
%intensity-frequency data.  Therefore, each index value increments by 1/15
%Hz
MinFreq = 0.3;
MaxFreq = 7;
Window = 2000;
%Perform a fourier transform over a moving region of interest
%From this transform, extract the frequency of the breathing peak.
%The breathing peak is found by finding the tallest peak in a region of
%interest selected based on manual measurement of breathing frequency
BreathFreq = ones(2,(m-Q));
BreathAmp = ones(2,(m-Q));

for iii = 1:3
for j = 1:(m-Q)
    Freq1 = abs(real(fft(smooth(XPos(iii,j:(j+Window))))));
    Freq2 = abs(real(fft(smooth(YPos(iii,j:(j+Window))))));
    [BreathAmp(2*iii-1,j), BreathFreq(2*iii-1,j)] =
max(smooth(Freq1(round(MinFreq/15*length(Freq1)/2):round(MaxFreq/15*length(Fr
eq1)/2)),1));
    [BreathAmp(2*iii,j), BreathFreq(2*iii,j)] =
max(smooth(Freq2(round(MinFreq/15*length(Freq2)/2):round(MaxFreq/15*length(Fr
eq2)/2)),1));
    figure(55)
    hold on

plot(linspace(MinFreq,MaxFreq,length(Freq1(round(MinFreq/15*length(Freq1)/2):
round(MaxFreq/15*length(Freq1)/2)))),Freq1(round(MinFreq/15*length(Freq1)/2):
round(MaxFreq/15*length(Freq1)/2)).^2)
end
figure(5)
hold on
plot((1:length(BreathFreq(2*iii-1,:)))/30,MinFreq + smooth(BreathFreq(2*iii-
1,:),30)./(Window/2).*15);
plot((1:length(BreathFreq(2*iii,:)))/30,MinFreq +
smooth(BreathFreq(2*iii,:),30)./(Window/2).*15);
xlabel('Time, s')
ylabel('Breath Frequency, Hz')
figure(6)
clf
hold on
plot((1:length(BreathAmp(2*iii-1,:)))/30,smooth(BreathAmp(2*iii-1,:),5));
plot((1:length(BreathAmp(2*iii,:)))/30,smooth(BreathAmp(2*iii,:),5));
xlabel('Time, s')
ylabel('Relative Breathing Amplitude')
end
meanfreq = zeros(1,length(BreathFreq(1,:)));
for v = 1:6
    meanfreq = meanfreq + BreathFreq(v,:);
end
figure(99)
meanfreq = meanfreq/6;
plot((1:length(BreathFreq(iii,:)))/30,MinFreq +
smooth(meanfreq,30)/(Window/2)*15);


%This is where we plot the frequency data after analysis
```

```
%Our frequency data is still recorded as an array index.  We convert this
%value to a frequency and filter noise first with a median filter to remove
%extreme changes and then a smoothing filter to mitigate noise.
figure(999)
plot((1:length(BreathFreq(1,:)))/30,smooth(medfilt1(BreathFreq(1,:)/(Window/2
)*15+MinFreq,30),800),'b')
xlabel('Time, s')
ylabel('Breathing Frequency, Hz')


end
```
