# Supplementary material for: Nesting box imager: Contact-free, real-time measurement of activity, surface body temperature, and respiratory rate applied to hibernating mouse models
Source: PLoS Biol. 2019 Jul 24;17(7):e3000406. doi: 10.1371/journal.pbio.3000406 (PMC6682158; doi:10.1371/journal.pbio.3000406)
Supplement: S3 Code — PIR, passive infrared. (PDF) [file pbio.3000406.s021.pdf]

```python
# Coded By Han Jong Shin, modified by WJI

from gpiozero import MotionSensor
from datetime import datetime
import time

#Button trigger; added this and writing of begin & end times 1.4.2018; WJI, note now have to hold button for longer than 0.5 sec
import RPi.GPIO as GPIO
GPIO.setmode(GPIO.BCM)
GPIO.setup(25, GPIO.IN, pull_up_down=GPIO.PUD_UP)

Txt = open("/media/pi/DATA/mousehouse/Motion.txt",'a')
Txt.write("Begin Time:\n")
filename = datetime.now().strftime("%m/%d/%Y %H:%M:%S\n")
Txt.write(filename)
Txt.write("Continue Experiment\n")
Txt.close()

pir = MotionSensor(4)
while True:
    input_state = GPIO.input(25)
    if input_state == False:
        Txt = open("/media/pi/DATA/mousehouse/Motion.txt",'a')
        Txt.write("End Time:\n")
        filename = datetime.now().strftime("%m/%d/%Y %H:%M:%S\n")
        Txt.write(filename)
        Txt.write("End Experiment\n")
        Txt.write("----------\n")
        Txt.close()
        time.sleep(0.5)
        quit()
    elif pir.motion_detected:
        Txt = open("/media/pi/DATA/mousehouse/Motion.txt",'a')
        filename = datetime.now().strftime("%m/%d/%Y %H:%M:%S 1\n") #1 = motion, dates formatted for ExpeData import after processing
        print(filename)
        #print(input_state)
        Txt.write(filename)
        Txt.close()
        time.sleep(0.5)
    else:  #added 2019-04-05 for motion sensor recording, prints when no motion detected
        Txt = open("/media/pi/DATA/mousehouse/Motion.txt",'a')
        filename = datetime.now().strftime("%m/%d/%Y %H:%M:%S 0\n") # 0 = no motion
        print(filename)
        Txt.write(filename)
        Txt.close()
        time.sleep(0.5)
```

ExpeData Macro for Processing Motion Data

```
[macro 1]
 ' Use this macro to process ADX and NBI motion data
create channel
assign title 7 = ADX_activity_mode_zero
assign title last_channel = ADX_activity_mode_zero
trans_corr_target channel 7    ADX_activity_mode_zero
transform remove_mode sd_multiplier 2.00 channel ADX_activity target ADX_activity_mode_zero
active channel ADX_activity_mode_zero
select selectedwindow from  15981 to  40799 of  43201
findwindow level selected width 50
select selectedwindow selected
correction zero_channel selected
active channel ADX_activity_mode_zero
correction boolean all_samples if data < 0 intercept 0 slope 0
create channel
assign title 8 =ADX_activity_m_z_int
assign title last_channel = ADX_activity_m_z_int
trans_corr_target channel 8    ADX_activity_m_z_int
transform integrate all_samples timebase seconds
active channel Motion_Cage1
create channel
assign title 9 = Motion_Cage1_clip
assign title last_channel = Motion_Cage1_clip
trans_corr_target channel 9 Motion_Cage1_clip
correction boolean all_samples if data < 0.5 intercept 0 slope 0
correction boolean all_samples if data > 1 intercept 1 slope 0
active channel Motion_Cage2
create channel
assign title 10 =Motion_Cage2_clip
assign title last_channel = Motion_Cage2_clip
trans_corr_target channel 10 Motion_Cage2_clip
correction boolean all_samples if data < 0.5 intercept 0 slope 0
correction boolean all_samples if data > 1 intercept 1 slope 0
create channel
assign title 11 = Motion_All
assign title last_channel = Motion_All
trans_corr_target channel 11 Motion_All
transform general all_samples expression C6+C9+C10
active channel Motion_All
create channel
assign title 12 = Motion_All_int
assign title last_channel = Motion_All_int
trans_corr_target channel 12 Motion_All_int
transform integrate all_samples timebase seconds
active channel NBI_motion_3
create channel
assign title 13 = NBI_motion_3_int
assign title last_channel = NBI_motion_3_int
```

active channel NBI_motion_3_int
trans_corr_target channel 13 NBI_motion_3_int
transform integrate all_samples timebase seconds
active channel Motion_Cage1_clip
create channel
assign title 14 = Motion_Cage_All
assign title last_channel = Motion_Cage_All
trans_corr_target channel 14 Motion_Cage_All
transform general all_samples expression C9+C10
active channel Motion_Cage_All
create channel
assign title 15 = Motion_Cage_All_int
assign title last_channel = Motion_Cage_All_int
trans_corr_target channel 15 Motion_Cage_All_int
transform integrate all_samples timebase seconds
active channel Motion_Cage_All_int
save all_samples sscf
[/macro 1]
ast_channel = Motion_All_int
trans_corr_target channel 10 Motion_All_int
transform integrate all_samples timebase seconds
active channel Motion_All_int
active channel NBI_motion_3
create channel
assign title 11 = NBI_motion_int
assign title last_channel = NBI_motion_int
[/macro 1]
ADX_activity_m_z_int Motion_Cage1_clip Motion_Cage2_clip Motion_All Motion_All_int NBI_motion_3_int
[/macro 1]
ast_channel = Motion_All_int
trans_corr_target channel 10 Motion_All_int
transform integrate all_samples timebase seconds
active channel Motion_All_int
active channel NBI_motion_3
create channel
assign title 11 = NBI_motion_int
assign title last_channel = NBI_motion_int
[/macro 1]
